# Psychological and pharmacological interventions for social anxiety disorder in adults: a systematic review and network meta-analysis

**DOI:** 10.1016/S2215-0366(14)70329-3

**Published:** 2014-09-26

**Authors:** Evan Mayo-Wilson, Sofia Dias, Ifigeneia Mavranezouli, Kayleigh Kew, David M Clark, A E Ades, Stephen Pilling

**Affiliations:** a Centre for Outcomes Research and Effectiveness, Research Department of Clinical, Educational, and Health Psychology, University College London, London, UK; b School of Social and Community Medicine, University of Bristol, Bristol, UK; c Population Health Research Institute, St George's, University of London, London, UK; d Department of Experimental Psychology, University of Oxford, Oxford, UK

## Abstract

**Background:**

Social anxiety disorder—a chronic and naturally unremitting disease that causes substantial impairment—can be treated with pharmacological, psychological, and self-help interventions. We aimed to compare these interventions and to identify which are most effective for the acute treatment of social anxiety disorder in adults.

**Methods:**

We did a systematic review and network meta-analysis of interventions for adults with social anxiety disorder, identified from published and unpublished sources between 1988 and Sept 13, 2013. We analysed interventions by class and individually. Outcomes were validated measures of social anxiety, reported as standardised mean differences (SMDs) compared with a waitlist reference. This study is registered with PROSPERO, number CRD42012003146.

**Findings:**

We included 101 trials (13 164 participants) of 41 interventions or control conditions (17 classes) in the analyses. Classes of pharmacological interventions that had greater effects on outcomes compared with waitlist were monoamine oxidase inhibitors (SMD −1·01, 95% credible interval [CrI] −1·56 to −0·45), benzodiazepines (−0·96, −1·56 to −0·36), selective serotonin-reuptake inhibitors and serotonin–norepinephrine reuptake inhibitors (SSRIs and SNRIs; −0·91, −1·23 to −0·60), and anticonvulsants (−0·81, −1·36 to −0·28). Compared with waitlist, efficacious classes of psychological interventions were individual cognitive–behavioural therapy (CBT; SMD −1·19, 95% CrI −1·56 to −0·81), group CBT (−0·92, −1·33 to −0·51), exposure and social skills (−0·86, −1·42 to −0·29), self-help with support (−0·86, −1·36 to −0·36), self-help without support (−0·75, −1·25 to −0·26), and psychodynamic psychotherapy (−0·62, −0·93 to −0·31). Individual CBT compared with psychological placebo (SMD −0·56, 95% CrI −1·00 to −0·11), and SSRIs and SNRIs compared with pill placebo (−0·44, −0·67 to −0·22) were the only classes of interventions that had greater effects on outcomes than appropriate placebo. Individual CBT also had a greater effect than psychodynamic psychotherapy (SMD −0·56, 95% CrI −1·03 to −0·11) and interpersonal psychotherapy, mindfulness, and supportive therapy (−0·82, −1·41 to −0·24).

**Interpretation:**

Individual CBT (which other studies have shown to have a lower risk of side-effects than pharmacotherapy) is associated with large effect sizes. Thus, it should be regarded as the best intervention for the initial treatment of social anxiety disorder. For individuals who decline psychological intervention, SSRIs show the most consistent evidence of benefit.

**Funding:**

National Institute for Health and Care Excellence.

## Introduction

Social anxiety disorder, or social phobia, affects 7% of the population^1^ and follows a chronic and debilitating course if untreated.^2^ Findings from meta-analyses suggest that the disorder responds well to pharmacological,^3^ psychological,^4^ and self-help interventions,^5^ but most reviews have been limited to pairwise comparisons of subsets of these interventions.

Network meta-analysis has the advantage that all interventions that have been tested in randomised controlled trials (RCTs) can be simultaneously compared and their effects can be estimated relative to each other and to a common reference condition (eg, waitlist). Estimates of the effects of pairs of treatments that have often, rarely, or never been directly compared in a RCT can be calculated. As a consequence, network meta-analysis overcomes some of the limitations of traditional meta-analysis, in which conclusions are largely restricted to comparisons between treatments that have been directly compared in RCTs.

We undertook a network meta-analysis of all psychological and pharmacological interventions that are used in routine clinical practice for the initial treatment of social anxiety disorder and have been tested in RCTs.

## Methods

### Search strategy and selection criteria

We did a systematic review of interventions for social anxiety disorder according to Preferred Reporting Items for Systematic reviews and Meta-Analyses (PRISMA) guidelines.^6^ We searched the following databases between 1988 and Sept 13, 2013, with no language limits set, for published and unpublished studies on treatment of adults with social anxiety disorder: Australian Education Index, Allied and Complementary Medicine Database, Applied Social Services Index and Abstracts, British Education Index, Cochrane Database of Systematic Reviews, CENTRAL, Cumulative Index to Nursing and Allied Health Literature, Database of Abstracts of Reviews and Effectiveness, Embase, Education Resources in Curriculum, Health Management Information Consortium, Health Technology Assessment, International Bibliography of Social Science, Medline, PreMEDLINE, PsycBOOKS, PsycEXTRA, PsycINFO, Sociological Abstracts, Social Services Abstracts, and Social Sciencies Citation Index (appendix A). We also searched trial registries and reference lists of reviews and included studies. We consulted a group of experts from the National Institute for Health and Care Excellence (NICE) Guideline Development Group to identify relevant studies. We also wrote to authors of included studies to request trial registration details and unpublished outcomes and data; we also asked them to identify other potentially relevant studies.

All citations were screened by one author (KK or EM-W) who excluded citations that were not related to trials or to social anxiety disorder; potentially relevant citations were checked independently by a second author (EM-W or KK). Study characteristics, outcomes, and risk of bias^7^ were extracted by one author (KK or EM-W) and checked independently by a second (EM-W or KK).

Randomised clinical trials of interventions for adults aged at least 18 years who fulfilled diagnostic criteria for social anxiety disorder were included. Studies that primarily focused on the treatment of comorbid disorders (eg, substance abuse) were excluded, but participants in the included studies often met criteria for another disorder (eg, depression) and were included. Eligible interventions were oral drugs (fixed or flexible doses), psychological or behavioural interventions (eg, promotion of exercise; panel), and combinations of interventions. Pharmacological interventions did not need to be licensed for social anxiety disorder, but interventions not used routinely in the treatment of social anxiety disorder, according to the consensus of the investigators and the NICE Guideline Development Group for the guideline *Social anxiety disorder: recognition, assessment and treatment*, were excluded (ie, exposure with a cognitive enhancer, surgical interventions, injected drugs, and antipsychotics). Studies of computerised cognitive bias modification were analysed in a separate review (unpublished). We excluded drugs that are no longer marketed (eg, brofaromine) if trials compared them only with placebo because these trials would not provide information about eligible interventions.

Panel **Definition of psychological interventions**

**Promotion of exercise**

Behavioural change programmes that promote increased physical activity.

**Exposure and social skills**

Behavioural interventions that involve systematic exposure to social interactions or public speaking, but that do not include explicit cognitive techniques.

**Group CBT**

Therapist-led, group-based interventions that use both behavioural strategies (eg, exposure) and various cognitive strategies (eg, cognitive restructuring, video feedback, and attention training). Specific CBT manuals were followed for this intervention or the study investigators described the intervention as CBT.

**Individual CBT**

Individual interventions for which specific CBT manuals were followed or that were described as CBT by study investigators.

**Other psychological therapy**

Psychological therapies not included elsewhere were grouped to improve estimates of variance for the class model. This class includes the specific effects of interpersonal psychotherapy, mindfulness training, and supportive therapy.

**Psychodynamic psychotherapy**

Short-term psychodynamic psychotherapy, for which a treatment manual specifically for social anxiety disorder can be followed.

**Psychological placebo**

A psychological intervention that includes features common to most well-undertaken psychological therapies (ie, non-specific components of treatment) and that was designed as a credible intervention.

**Self-help with support**

Interventions (usually CBT based) that are delivered by book or computer with limited therapist support (eg, short meetings, email support, or phone calls). For the purpose of clinical trials, participants typically received clinical interviews at the beginning and end of treatment.

**Self-help without support**

Interventions (usually CBT based) that are delivered exclusively by book or computer. For the purpose of clinical trials, participants were interviewed at the beginning and end of treatment.

CBT=cognitive–behavioural therapy.

We limited the network meta-analysis to interventions that people with social anxiety disorder and clinicians might regard as first-line treatments because network analysis assumes that treatment effects are transferable across studies. Ideally, all trial populations included in the network meta-analysis could have been eligible for all the treatment options investigated. Clinically, people choosing a first-line intervention have a different set of treatment options compared with people choosing second-line interventions; there would be a high risk that the assumption of exchangeability would be violated by the inclusion of clinically heterogeneous populations (eg, people who had not responded to treatments assessed in other studies). We identified eligible interventions by reviewing published and unpublished studies and through consultation with clinicians and experts (including people with social anxiety disorder, pharmacists, psychologists, and psychiatrists). We included interventions rather than excluded them if some experts thought they could be used as a first-line treatment.

### Statistical analysis

If a study reported continuous results for participants who completed the study only, as well as continuous results that accounted for missing data (eg, effects calculated using multiple imputation), we extracted the data that accounted for missing data. Studies reported several measures of social anxiety, none of which were common to all trials, so we calculated treatment effects for each study as a standardised mean difference (SMD). To reduce measurement error, we calculated the mean effect (Hedges' *g*) of all eligible scales for studies that reported more than one measure, taking between-scale correlation into account.^8^ For trials that reported only the change from baseline, the SD at baseline was used to ensure standardising constants were comparable across trials. Based on published psychometric properties and data from clinically referred participants who completed several measures (appendix A), we assumed that measures were equally responsive and had a mean correlation of 0·65.

Where reported, we also extracted data for recovery from social anxiety disorder (ie, no longer meeting criteria for the diagnosis) assuming that study dropouts had not recovered. We used the relation between continuous outcomes and recovery to estimate the treatment effect for all studies, including those that did not report recovery (appendix A).

We did a Bayesian random-effects network meta-analysis,^9^ which accounts for the correlation between trial-specific effects and random effects of trials with more than two arms.^10^ We analysed interventions by class (eg, selective serotonin-reuptake inhibitors and serotonin-norepinephrine reuptake inhibitors [SSRIs and SNRIs]) and individually (eg, sertraline). In general, treatments with similar mechanisms of action were grouped in classes in which pooled effects were assumed to be similar. This grouping had the effect of drawing individual treatment effects towards the class mean. We used non-informative priors, except for the prior for within-class variability. Because there were few data to reliably estimate within-class variation, this prior was informative and was restricted with an inverse-gamma prior. This restriction limited variability to a clinically plausible range and had the effect of restricting the effect of outliers within a class; specific interventions with inconsistent results based on limited data would have otherwise had an undue effect on the results. For treatments not belonging to a class, we assumed no class variability and estimated only between-study heterogeneity. Combination interventions were included in a class because analysing of each combination as a distinct class would underestimate true variance (appendix A).

We estimated the effect for each class and for each individual intervention using Markov chain Monte Carlo implemented in WinBUGS version 1.4.3.^11^ The first 20 000 iterations were discarded, and 50 000 further iterations were run. Two chains with different initial values were run simultaneously to assess convergence using the Gelman–Rubin diagnostic trace plots. We estimated effects with and without the consistency assumptions for individual treatment effects (ie, without grouping by class) and compared the residual deviance of each to assess consistency.^12^ We compared the fit of the standard model to the class model by comparing the residual deviance, and we chose the model with the lowest deviance information criterion.^9^ We used treatment effects to estimate change on continuous measures and the absolute rate of recovery for each intervention with 95% credible intervals (CrIs). Main effects are reported compared with waitlist, which was chosen as the reference treatment a priori.

All outcomes and study effects used in the analysis are available online (appendix B).

This study is registered with PROSPERO, number CRD42012003146.

### Role of the funding source

NICE commissioned the National Collaborating Centre for Mental Health (NCCMH) to develop guidance for the identification and management of social anxiety disorder. NICE also approved funding for the Technical Support Unit to support NCCMH in undertaking a network meta-analysis of intervention studies.

The funder of the study had no further role in study design, data collection, data analysis, data interpretation, or writing of the report. All authors had full access to all the data in the study and had final responsibility for the decision to submit for publication.

## Results

**Figure 1:**
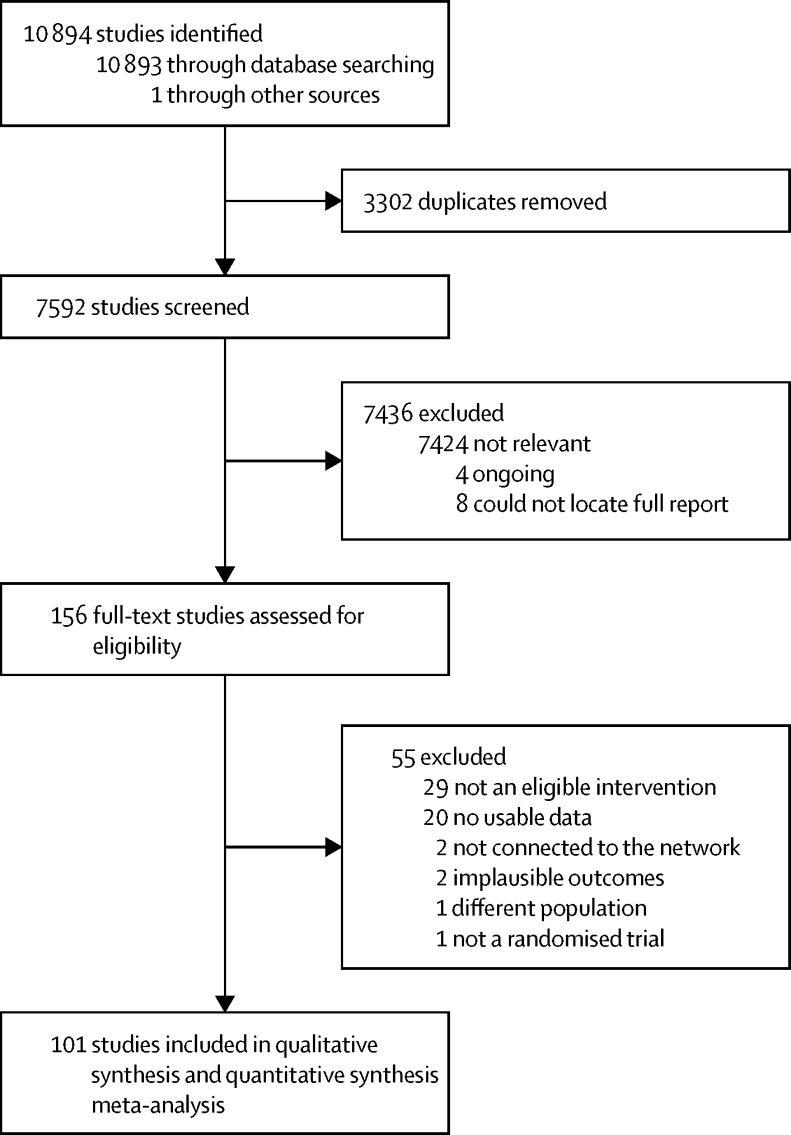
PRISMA flowchart PRISMA=Preferred Reporting Items for Systematic reviews and Meta-Analyses.

Between 1988 and Sept 13, 2013, we identified 168 potentially eligible studies, 12 of which were excluded: four were ongoing studies and for eight studies we could not identify a complete study report. We assessed 156 studies for eligibility (figure 1). 55 studies were excluded (appendix A) because they did not include an eligible intervention (n=29), reported no usable data (n=20), included no intervention already in the analysis and thus were not connected to this network (n=2), reported implausible outcomes (n=2), included a different population (n=1), or were not a randomised trial (n=1). 101 studies were included in the network analysis (appendix A).

14 229 participants were randomly assigned in the trials, and 13 164 were included in the analysis because some trials did not report outcomes for all participants. There were 18–839 participants per study. Trials assessed 41 interventions or control conditions, which were grouped into 17 classes. Most trials included two groups (n=64), but some included three (n=28), four (n=7), or five groups (n=2). The median and mean duration of treatment was 12 weeks (range 2–28 weeks). Few studies provided controlled results for long-term follow-up, and so long-term follow-up data were not included in our analyses.

Participants had severe and longstanding social anxiety; of 65 studies reporting baseline Liebowitz Social Anxiety Scale^13^ scores, the median of means was 78 (appendix A). The median of means age was 36 years and the median of percentages of participants who were white was 80%. About half of the included participants were women (52% median of means). Most psychological studies did not exclude participants receiving drug treatment, but trials of psychological interventions generally required participants to be on a stable dose of drug treatment for several months before random allocation. Participants were not receiving drug treatment in 44 trials. In 27 trials, 27% of participants (median of means) were receiving drug treatment at randomisation. The demographic characteristics of participants were similar across comparisons (appendix A), and there were no obvious differences in the initial severity of social anxiety symptoms; variation in severity was limited because studies had similar inclusion criteria.

We assessed all included trials for risk of bias (appendix A). Sequence generation and allocation concealment were adequately described in 74 and 69 trials, respectively (appendix A). Trials of psychological interventions were regarded as at high risk of bias for participant and provider masking per se, although treatment effects and side-effects could also make maintenance of masking difficult in pharmacological trials. Most reported outcomes were self-rated, and assessors were aware of treatment assignment in five trials. For incomplete outcome data, 26 trials were at high risk of bias (eg, those that reported only completer analyses and those with lots of missing data), and how missing data were handled was unclear in four trials.

Most included trials were not registered; only 37 trials were at low risk of selective outcome reporting bias (appendix A). In addition to risk of selective outcome reporting for included studies, there is risk of reporting bias because we could not locate a full report for eight studies, 20 studies reported no usable data, and two studies reported implausible outcomes. Results can be overestimated as a result of publication bias, particularly for interventions developed before mandatory trial registration. Unpublished information was obtained from trial investigators for 34 studies, including unpublished outcomes for 22 trials.

Excluding masking of participants and providers, which was impossible in studies of psychological interventions and difficult to maintain in studies of pharmacological interventions, only 28 trials were at low risk of bias for all other domains assessed by the Cochrane risk of bias assessment (appendix A).

**Figure 2:**
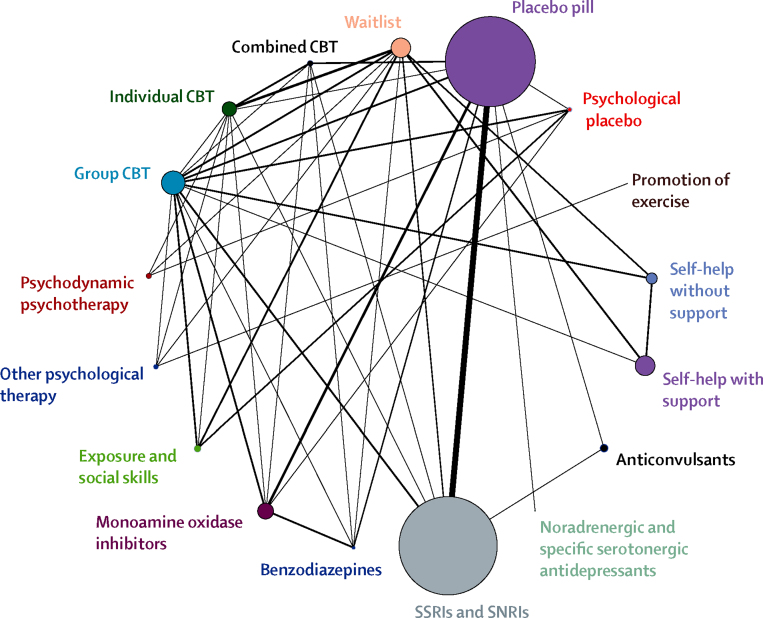
Network diagram representing direct comparisons among classes The width of lines represents the number of trials in which each direct comparison is made. The size of each circle represents the number of people who received each treatment. CBT=cognitive–behavioural therapy. SNRI=serotonin–norepinephrine reuptake inhibitor. SSRI=selective serotonin-reuptake inhibitor.

Figure 2 shows the network of comparisons among classes. Of 820 possible comparisons among 41 intervention or control conditions, 84 were studied directly (appendix A). 76 studies compared interventions with a control group; most drugs were compared with placebo, and most psychological interventions were compared with waitlist or with psychological placebo. The network also included 58 studies that compared active interventions, including four studies that compared psychological with pharmacological interventions.

25 trials also reported recovery (appendix A), and we compared effects for continuous measures and loss of diagnosis for these studies, which suggested that continuous values provide lower treatment effects compared with odds ratios of recovery.

There was potential for inconsistency in nine of the 44 loops in the network—others were formed by multi-arm trials that are consistent by definition. There were no substantial differences in magnitude and direction between the results of the network meta-analysis and the results of pairwise comparisons. The posterior mean of the residual deviance was 165·3 in the standard network meta-analysis model compared with 176·3 in the independent-effect model that compares favourably with the number of treatment groups (n=148), suggesting the network better estimates treatment effects than pairwise analyses alone with no evidence of inconsistency.^9^

The random-effects class model was a good fit to the data compared with the individual-effects model (deviance information criterion 364·8 *vs* 371·0; lower values suggest a better fit), although the between-trials SD for heterogeneity had a posterior median of 0·19 (95% CrI 0·14–0·25). That is, there was some variability between classes that might be attributable to differences among the individual treatments beyond the within-class variability. For classes with few members, there was little information about within-class variability and the prior for within-class variability led to increased uncertainty in the estimated class effects.

**Figure 3:**
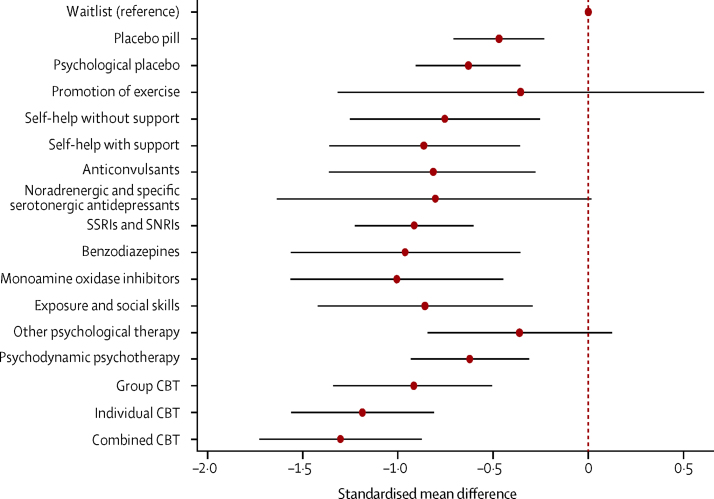
Effect of each class of intervention compared with waitlist Data are standardised mean difference and 95% credible intervals compared with waitlist as a reference. CBT=cognitive–behavioural therapy. SNRI=serotonin–norepinephrine reuptake inhibitor. SSRI=selective serotonin-reuptake inhibitor.

All pharmacological interventions apart from noradrenergic and specific serotonergic antidepressants had greater effects on outcomes compared with waitlist (table; figure 3). Mirtazapine, a noradrenergic and sepcific serotonergic antidepressant, was the only pharmacological intervention in a class by itself; its effect was not greater than that for waitlist (class effect SMD −0·80, 95% CrI −1·64 to 0·01), but only 30 people received the intervention. The largest effects were for MAOIs (class effect SMD −1·01, 95% CrI −1·56 to −0·45) and benzodiazepines (−0·96, −1·56 to −0·36), but the evidence for these effects was limited compared with evidence for SSRIs and SNRIs (−0·91, −1·23 to −0·60); more people received SSRIs and SNRIs (n=4043) than all other pharmacological interventions (n=999) or all psychological interventions (n=3312).

**Table:** Summary of treatment effects compared with waitlist

		**Trials**	**Participants**	**Class effect SMD (95% CrI)**	**Individual effect SMD (95% CrI)**
**Controls**					
Waitlist		28	802	Reference	Reference
Placebo pill		42	3623	−0·47 (−0·71 to −0·23)	..
Psychological placebo		6	145	−0·63 (−0·90 to −0·36)	..
**Pharmacological interventions**					
Anticonvulsants		5	242	−0·81 (−1·36 to −0·28)	..
	Gabapentin	1	34	..	−0·89 (−1·42 to −0·37)
	Levetiracetam	1	9	..	−0·83 (−1·50 to −0·18)
	Pregabalin	3	199	..	−0·72 (−1·07 to −0·37)
Benzodiazepines		5	112	−0·96 (−1·56 to −0·36)	..
	Alprazolam	1	12	..	−0·85 (−1·40 to −0·30)
	Clonazepam	4	100	..	−1·07 (−1·44 to −0·70)
Monoamine oxidase inhibitors		11	615	−1·01 (−1·56 to −0·45)	..
	Moclobemide	6	490	..	−0·74 (−1·03 to −0·44)
	Phenelzine	5	125	..	−1·28 (−1·57 to −0·98)
Noradrenergic and specific serotonergic antidepressants (mirtazapine)		1	30	−0·80 (−1·64 to 0·01)	−0·81 (−1·45 to −0·16)
SSRIs and SNRIs		32	4043	−0·91 (−1·23 to −0·60)	..
	Citalopram	2	18	..	−0·83 (−1·28 to −0·39)
	Escitalopram	2	675	..	−0·88 (−1·20 to −0·56)
	Fluoxetine	3	107	..	−0·87 (−1·16 to −0·57)
	Fluvoxamine	5	500	..	−0·94 (−1·25 to −0·63)
	Paroxetine	12	1449	..	−0·99 (−1·26 to −0·73)
	Sertraline	3	535	..	−0·92 (−1·23 to −0·61)
	Venlafaxine	5	759	..	−0·96 (−1·25 to −0·67)
**Psychological and behavioural interventions**					
Exercise promotion		1	18	−0·36 (−1·32 to 0·61)	−0·36 (−1·07 to 0·36)
Exposure and social skills		10	227	−0·86 (−1·42 to −0·29)	..
	Exposure in vivo	9	199	..	−0·83 (−1·07 to −0·59)
	Social skills training	1	28	..	−0·88 (−1·38 to −0·38)
Group CBT		28	984	−0·92 (−1·33 to −0·51)	..
	Heimberg model	11	338	..	−0·80 (−1·02 to −0·58)
	Other (no model specified)	16	583	..	−0·85 (−1·04 to −0·68)
	Enhanced CBT	1	63	..	−1·10 (−1·49 to −0·71)
Individual CBT		15	562	−1·19 (−1·56 to −0·81)	..
	Hope, Heimberg, and Turk model	2	53	..	−1·02 (−1·42 to −0·62)
	Other (no model specified)	6	163	..	−1·19 (−1·48 to −0·89)
	Clark and Wells cognitive therapy model	3	97	..	−1·56 (−1·85 to −1·27)
	Clark and Wells cognitive therapy shortened sessions	4	249	..	−0·97 (−1·21 to −0·74)
Other psychological therapy		7	182	−0·36 (−0·84 to 0·12)	..
	Interpersonal psychotherapy	2	64	..	−0·43 (−0·83 to 0·04)
	Mindfulness training	3	64	..	−0·39 (−0·82 to 0·03)
	Supportive therapy	2	54	..	−0·26 (−0·72 to 0·20)
Psychodynamic psychotherapy		3	185	−0·62 (−0·93 to −0·31)	..
Self-help with support		16	748	−0·86 (−1·36 to −0·36)	..
	Book with support	3	52	..	−0·85 (−1·17 to −0·53)
	Internet with support	13	696	..	−0·88 (−1·04 to −0·71)
Self-help without support		9	406	−0·75 (−1·25 to −0·26)	..
	Book without support	4	136	..	−0·84 (−1·08 to −0·60)
	Internet without support	5	270	..	−0·66 (−0·94 to −0·39)
**Combined interventions**					
Combined		5	156	−1·30 (−1·73 to −0·88)	..
	Group CBT and moclobemide	1	22	..	−1·23 (−1·72 to −0·74)
	Group CBT and fluoxetine	1	59	..	−0·95 (−1·34 to −0·58)
	Group CBT and phenelzine	1	32	..	−1·69 (−2·10 to −1·27)
	Psychodynamic and clonazepam	1	29	..	−1·28 (−1·82 to −0·74)
	Paroxetine and clonazepam	1	14	..	−1·35 (−1·93 to −0·79)

CBT=cognitive–behavioural therapy. CrI=credible interval. SMD=standardised mean difference. SNRI=serotonin–norepinephrine reuptake inhibitor. SSRI=selective serotonin-reuptake inhibitor.

All psychological interventions apart from promotion of exercise and other psychological therapies (supportive therapy, mindfulness, and interpersonal psychotherapy) had greater effects on outcomes than did waitlist (table; figure 3). In decreasing order of effect size, these were individual cognitive–behavioural therapy (CBT; class effect SMD −1·19, 95% CrI −1·56 to −0·81), group CBT (−0·92, −1·33 to −0·51), exposure and social skills (−0·86, −1·42 to −0·29), self-help with support (−0·86, −1·36 to −0·36), self-help without support (−0·75, −1·25 to −0·26), and psychodynamic psychotherapy (−0·62, −0·93 to −0·31).

**Figure 4:**
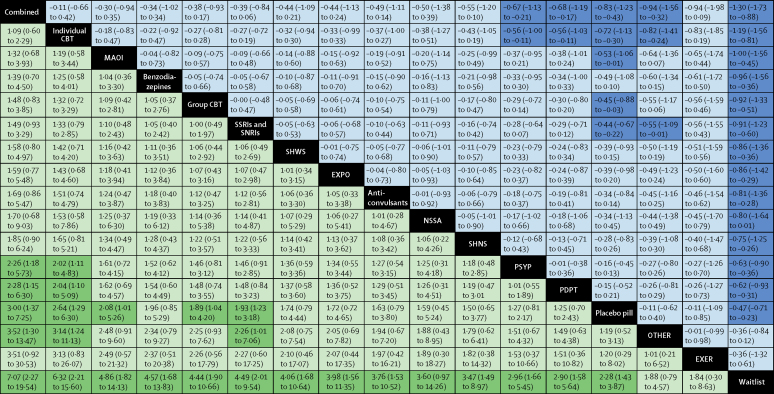
Efficacy of classes of interventions Classes of interventions are ordered according to efficacy ranking from largest mean effect (top, left) to smallest mean effect (bottom, right). Data in blue represent the effects on symptoms of social anxiety (SMD [95% CrI]); SMD less than 0 favours the intervention in the row. Data in green represent the effects on recovery (RR [95% CrI]); RR greater than 1 favours the intervention in the column. Significant results are shaded dark blue and dark green. CBT=cognitive–behavioural therapy. CrI=credible interval. EXER=promotion of exercise. EXPO=exposure and social skills. MAOI=monoamine oxidase inhibitors. NSSA=noradrenergic and specific serotonergic antidepressants. OTHER=other psychological therapy. PDPT=psychodynamic psychotherapy. PSYP=psychological placebo. RR=risk ratio. SHNS=self-help without support. SHWS=self-help with support. SMD=standardised mean difference. SNRI=serotonin–norepinephrine reuptake inhibitors. SSRI=selective serotonin-reuptake inhibitor.

Compared with pill placebo, MAOIs (SMD −0·53, 95% CrI −1·06 to −0·01) and SSRIs and SNRIs (−0·44, −0·67 to −0·22) had greater effects on outcomes, and pill placebo itself had a greater effect than waitlist (−0·47, −0·71 to −0·23; figure 4). Of the psychological interventions, only individual CBT had a greater effect on outcomes than psychological placebo (SMD −0·56, 95% CrI −1·00 to −0·11). Individual CBT also had a greater effect than pill placebo (SMD −0·72, 95% CrI −1·13 to −0·30), psychodynamic psychotherapy (−0·56, −1·00 to −0·11), and other therapies (−0·82, −1·41 to −0·24; figure 4). Figure 4 also expresses these treatment effects on the probability of recovery (ie, no longer meeting criteria for diagnosis).

Of the pharmacological interventions, there were greater individual effects compared with waitlist for all SSRIs (citalopram, escitalopram, fluoxetine, fluvoxamine, paroxetine, and sertraline) and the SNRI venlafaxine. Effects of SSRIs and SNRIs were measured in 32 studies, and they were similar in magnitude within the class except for citalopram, which was assessed in two small studies; all individual SMDs were within 0·08 of the class SMD. Compared with waitlist, the effects of the MOAIs phenelzine (SMD −1·28, −1·57 to −0·98) and moclobemide (−0·74, −1·03 to −0·44) were also greater; however, only 125 people received phenelzine across five trials and the results might be overestimated. The large effect for phenelzine was dissimilar to the small effect for moclobemide (appendix B), which was the only other MAOI included in the analysis.

The most efficacious psychological interventions were individual CBT—following the Clark and Wells model (SMD −1·56, 95% CrI −1·85 to −1·27),^14^ the Hope, Heimberg and Turk model (−1·02, −1·42 to −0·62),^15^ and CBT not following a named manual (−1·19, −1·48 to −0·89)—and group enhanced CBT (−1·10, −1·49 to −0·71; table). Supported self-help was efficacious when provided via the internet (SMD −0·88, 95% CrI −1·04 to −0·71) or by book (−0·85, −1·17 to −0·53). Psychological placebo also had a greater effect than waitlist (SMD −0·63, 95% CrI −0·90 to −0·36), and its effect was comparable to psychodynamic psychotherapy (−0·62, −0·93 to −0·31).

Several drugs had greater effects on outcomes compared with pill placebo: clonazepam, escitalopram, fluoxetine, fluvoxamine, moclobemide, paroxetine, phenelzine, sertraline, and venlafaxine (appendix B). Citalopram was the only included SSRI that did not have a greater effect than placebo. Of the psychological interventions, only Clark and Wells cognitive therapy model, Clark and Wells cognitive therapy model with shortened sessions, individual CBT, and group enhanced CBT had greater effects than psychological placebo. There was no consistent evidence of differential efficacy within pharmacotherapies. There was some evidence of differential efficacy within the psychological interventions. Individual CBT according to the Clark and Wells manual showed the most consistent evidence of greater effects, as suggested by non-overlapping 95% CrIs between this intervention and most other psychological intervention (table).

Combined interventions had greater effects on outcomes than waitlist overall (SMD −1·30, 95% CrI −1·73 to −0·88; table; figure 4), but the quality of the evidence was poor. Five different combinations of psychological and pharmacological interventions were assessed in one trial each; all reported large effects, but only 156 participants received combined interventions across all five trials. There was no evidence that combined interventions had greater effects than the leading monotherapies (table).

## Discussion

To our knowledge, this is the first time that psychological and pharmacological interventions for a mental health problem have been compared in network meta-analysis.^16^ The findings confirm that social anxiety disorder responds well to treatment, although many people continue to experience some symptoms after the end of the acute treatment phase.

Several classes of pharmacological and psychological intervention had greater effects on outcomes than did waitlist. Individual CBT and the class including SSRIs and SNRIs also had greater effects on outcomes than appropriate placebos, suggesting that they have specific effects. Psychological and pill placebo had greater effects than waitlist; investigation of these effects suggests that non-specific factors might account for about half the total effects of individual CBT and SSRIs. Comparisons between psychological interventions revealed some evidence of differential effects. In particular, individual CBT had a greater effect than psychodynamic psychotherapy and other psychological therapies (interpersonal psychotherapy, mindfulness, and supportive therapy). Many of the psychological treatments with large effects were versions of CBT (individual, group, or self-help), suggesting that CBT might be efficacious in a range of formats. Psychodynamic psychotherapy was also effective, although its effects were similar to psychological placebo.

Because pharmacological and psychological interventions were both efficacious, a logical question to ask is whether combined interventions might be more helpful than either intervention alone. Although large effect sizes were noted with combined treatments, only a few small studies were included, and there was no evidence that any combination was more efficacious than the leading pharmacological or psychological monotherapy in that combination.

There was little evidence of differential efficacy within or between classes of drugs. In the case of SSRIs and SNRIs, this finding is consistent with data from a previous network analysis, which showed no differences in efficacy but differences in tolerability.^17^ In the absence of convincing evidence for differential efficacy, differences in tolerability and side-effects are particularly important in the choice of treatment. SSRIs and SNRIs with a short half-life (eg, paroxetine and venlafaxine) are associated with the greatest risk of discontinuation effects, including effects during the treatment period and after the end of treatment.^18^, ^19^ Some side-effects such as increased agitation^18^ and sexual dysfunction^20^ can be especially distressing for people with social anxiety disorder, particularly if these effects are unexpected or if they reinforce existing worries. These issues should be discussed with patients before starting drug treatment.

We were not able to investigate whether immediate treatment effects persist or diminish in the long term because most trials stopped at the end of treatment. Findings from studies that have addressed this issue^21^, ^22^ suggest that most people who respond to a SSRI will relapse within a few months if the drug is discontinued after acute treatment, and about 25% of people who respond to SSRI treatment and continue drug treatment will relapse within 6 months. By contrast, the effects of psychological interventions are generally well maintained at follow-up,^23^ and participants can continue to apply new skills and make further gains after the end of acute treatment.^24^ For this reason, and because of the lower risk of side-effects, psychological interventions should be preferred over pharmacological interventions for initial treatment.^25^

This study has several limitations. There were only a few studies of moderate size for several included interventions, and some have only been tested by one or two research groups. We included a broad range of interventions, which varied in duration, and there might be unknown differences among participants in different trials. However, we did not identify any systematic differences in participant demographics or initial symptom severity. Direct and indirect results were consistent, which provides further support to the pooled results. Control conditions were heterogeneous and rarely described in detail. Future trials should more clearly describe what was intended and what was actually received by people in control conditions.^26^, ^27^ Statistical power might have been limited because we used scores after treatment rather than the change in scores and because we calculated effects conservatively, estimating effects accounting for dropout (eg, using last-observation-carried-forward) where possible. Conversely, pairwise analyses of small studies sometimes overestimate effects compared with large studies.^28^, ^29^ Uncertainty in mean effects (ie, large CrIs) suggests that more research would improve our understanding of how these treatments compare. Specifically, large trials that compare active interventions and independent replications would improve the precision of these estimates and increase confidence in their external validity. We included only outcomes at the end of treatment; trials comparing active interventions with controlled long-term follow-up would provide better evidence of sustained effects.

Data for cost-effectiveness and side-effects both affect choices, and a cost-effectiveness analysis will be reported elsewhere. Taking these factors into account, NICE recently concluded that individual CBT should be offered as the treatment of choice for social anxiety disorder. For individuals who decline individual CBT, a SSRI is recommended for people who would prefer drug treatment and CBT-based supported self-help is recommended for people who prefer another psychological intervention. Psychodynamic psychotherapy is recommended as a third-line option, and other drugs are recommended only for people who do not respond to initial treatments.^25^, ^30^ Thus, NICE recommendations are consistent with the results of this study, which suggests that increased access to treatment would reduce disability and improve quality of life for people with social anxiety disorder.
