# Supplementary material for: Psychological and pharmacological interventions for social anxiety disorder in adults: a systematic review and network meta-analysis
Source: Lancet Psychiatry. 2014 Sep 26;1(5):368–76. doi: 10.1016/S2215-0366(14)70329-3 (PMC4287862; doi:10.1016/S2215-0366(14)70329-3)
Supplement: Supplementary appendix A [file mmc1.pdf]

THE LANCET Psychiatry

Supplementary appendix A

This appendix formed part of the original submission and has been peer reviewed. We post it as supplied by the authors.

Supplement to: Mayo-Wilson E, Dias S, Mavranezouli I, et al. Psychological and pharmacological interventions for social anxiety disorder in adults: a systematic review and network meta-analysis. *Lancet Psychiatry* 2014; published online Sept 26. http://dx.doi.org/10.1016/S2215-0366(14)70329-3.

INTERVENTIONS FOR SOCIAL ANXIETY DISORDER IN ADULTS

CONTENTS

Appendix 1: Search strategy
Appendix 2: outcome measures included
Appendix 3: Correlation among measures (change during treatment)
Appendix 4: Continuous effects compared with observed recovery
Appendix 5: WINBUGS code
Appendix 6: Characteristics of excluded studies, and references
Appendix 7: Characteristics of included studies, and references
Appendix 8: Baseline characteristics by comparison
Appendix 9: Risk of bias for each study
Appendix 10: Risk of bias summary
Appendix 11: Network of specific treatments, continuous outcomes
Appendix 12: Network of specific treatments, recovery
References

APPENDIX 1: SEARCH STRATEGY

OVID (Medline, PsycINFO, Embase)

1	avoidant personality disorder/ or hyperhidrosis/ or mutism/ or social phobia/
2	1 use emez
3	blushing/ or exp hyperhidrosis/ or mutism/ or phobic disorders/ or shyness/
4	3 use mesz, prem
5	avoidant personality disorder/ or elective mutism/ or social anxiety/ or social phobia/ or sweating/ or timidity/
6	5 use psyh
7	(((anxiet$ or anxious$ or phobia$ or phobic$) adj2 (performance or social$)) or socioanxi$ or sociophobi$ or ((blush$ or sweat$ or trembl$) adj3 (anxiet$ or anxious$ or chronic$ or excessiv$ or fear$ or severe)) or ((interpersonal or inter personal or social$ or socio$) adj2 (aversion$ or aversiv$ or confiden$ or difficult$ or disorder$ or distress$ or fear$)) or hyperhydrosis or hyperperspirat$ or (hyper adj (hydrosis or perspirat$)) or ((mute$ or mutism) adj2 (elective$ or selective$)) or ((negative evaluation or speak$) adj3 (anxiet$ or anxious$ or distress$ or fear$)) or paruresis or (((personalit$ or phobi$ or social$ or socio$) adj2 avoid$) or avoidant disorder) or (phobi$ adj2 neuros$) or phobic disorder$ or (school$ adj2 (anxiet$ or anxious$ or phobi$ or refuse or refusal)) or (shy or shyness) or specific phobia$).ti,ab.
8	or/2,4,6-7
9	(systematic review or meta analysis).sh.
10	9 use emez
11	meta analysis.sh,pt. or "review literature as topic"/
12	11 use mesz, prem
13	(literature review or meta analysis).sh,md. or systematic review.md.
14	13 use psyh
15	(exp bibliographic database/ or (((electronic or computer$ or online) adj database$) or bids or cochrane or embase or index medicus or isi citation or medline or psyclit or psychlit or scisearch or science citation or (web adj2 science)).ti,ab.) and (review$.ti,ab,sh,pt. or systematic$.ti,ab.)
16	15 use emez
17	(exp databases, bibliographic/ or (((electronic or computer$ or online) adj database$) or bids or cochrane or embase or index medicus or isi citation or medline or psyclit or psychlit or scisearch or science citation or (web adj2 science)).ti,ab.) and (review$.ti,ab,sh,pt. or systematic$.ti,ab.)
18	17 use mesz, prem
19	(computer searching.sh. or (((electronic or computer$ or online) adj database$) or bids or cochrane or embase or index medicus or isi citation or medline or psyclit or psychlit or scisearch or science citation or (web adj2 science)).ti,ab.) and (review$.ti,ab,pt. or systematic$.ti,ab.)
20	19 use psyh
21	((analy$ or assessment$ or evidence$ or methodol$ or qualitativ$ or quantativ$ or systematic$) adj2 (overview$ or review$)).tw. or ((analy$ or assessment$ or evidence$ or methodol$ or quantativ$ or qualitativ$ or systematic$).ti. and review$.ti,pt.) or (systematic$ adj2 search$).ti,ab.
22	(metaanal$ or meta anal$ or metasynthes$ or meta synthes$).ti,ab.
23	(research adj (review$ or integration)).ti,ab.
24	reference list$.ab.
25	bibliograph$.ab.
26	published studies.ab.
27	relevant journals.ab.
28	selection criteria.ab.
29	(data adj (extraction or synthesis)).ab.
30	(handsearch$ or ((hand or manual) adj search$)).ti,ab.
31	(mantel haenszel or peto or dersimonian or der simonian).ti,ab.
32	(fixed effect$ or random effect$).ti,ab.
33	((pool$ or combined or combining) adj2 (data or trials or studies or results)).ti,ab.
34	or/10,12,14,16,18,20-33
35	exp "clinical trial (topic)"/ or exp clinical trial/ or (crossover procedure or double blind procedure or placebo$ or randomization or random sample or single blind procedure).sh.
36	35 use emez
37	exp clinical trial/ or (cross-over studies or double-blind method or placebos or random allocation or randomization or single-blind method).sh. or (randomized controlled trials as topic/ and random$.sh.)
38	37 use mesz, prem
39	clinical trials/ or (placebo or random sampling).sh,id.
40	39 use psyh
41	(clinical adj2 trial$).ti,ab.
42	(crossover or cross over).ti,ab.

43 (((single$ or doubl$ or trebl$ or tripl$) adj2 blind$) or mask$ or dummy or singleblind$ or doubleblind$ or trebleblind$ or tripleblind$).ti,ab.
44 (placebo$ or random$).ti,ab.
45 (clinical trial$ or controlled clinical trial$ or random$).pt. or treatment outcome$.md.
46 animals/ not human$.mp.
47 animal$/ not human$/
48 (animal not human).po.
49 (or/36,38,40-45) not (or/46-48)
50 8 and (or/34,49)

CENTRAL
#1 mesh descriptor phobic disorders, this term only
#2 mesh descriptor hyperhidrosis explode all trees
#3 mesh descriptor mutism, this term only
#4 mesh descriptor blushing, this term only
#5 mesh descriptor shyness, this term only
#6 ((anxiet* or anxious* or phobia* or phobic*) near/2 (performance or social*)) or socioanxi* or sociophobi* or ((blush* or sweat* or trembl*) near/3 (anxiet* or anxious* or chronic* or excessiv* or fear* or severe)) or ((interpersonal or "inter personal" or social* or socio*) near/2 (aversion* or aversiv* or confiden* or difficult* or disorder* or distress* or fear*)) or hyperhydrosis or hyperperspirat* or (hyper near/1 (hydrosis or perspirat*)) or ((mute* or mutism) near/2 (elective* or selective*)) or (("negative evaluation" or speak*) near/3 (anxiet* or anxious* or distress* or fear*)) or paruresis or ((personalit* or phobi* or social* or socio*) near/2 avoid*) or "avoidant disorder" or (phobi* near/2 neuros*) or "phobic disorder*" or (school* near/2 (anxiet* or anxious* or phobi* or refuse or refusal)) or shy or shyness or "specific phobia*":ti 805
#7 ((anxiet* or anxious* or phobia* or phobic*) near/2 (performance or social*)) or socioanxi* or sociophobi* or ((blush* or sweat* or trembl*) near/3 (anxiet* or anxious* or chronic* or excessiv* or fear* or severe)) or ((interpersonal or "inter personal" or social* or socio*) near/2 (aversion* or aversiv* or confiden* or difficult* or disorder* or distress* or fear*)) or hyperhydrosis or hyperperspirat* or (hyper near/1 (hydrosis or perspirat*)) or ((mute* or mutism) near/2 (elective* or selective*)) or (("negative evaluation" or speak*) near/3 (anxiet* or anxious* or distress* or fear*)) or paruresis or ((personalit* or phobi* or social* or socio*) near/2 avoid*) or "avoidant disorder" or (phobi* near/2 neuros*) or "phobic disorder*" or (school* near/2 (anxiet* or anxious* or phobi* or refuse or refusal)) or shy or shyness or "specific phobia*":ab 1049
#8 ((anxiet* or anxious* or phobia* or phobic*) near/2 (performance or social*)) or socioanxi* or sociophobi* or ((blush* or sweat* or trembl*) near/3 (anxiet* or anxious* or chronic* or excessiv* or fear* or severe)) or ((interpersonal or "inter personal" or social* or socio*) near/2 (aversion* or aversiv* or confiden* or difficult* or disorder* or distress* or fear*)) or hyperhydrosis or hyperperspirat* or (hyper near/1 (hydrosis or perspirat*)) or ((mute* or mutism) near/2 (elective* or selective*)) or (("negative evaluation" or speak*) near/3 (anxiet* or anxious* or distress* or fear*)) or paruresis or ((personalit* or phobi* or social* or socio*) near/2 avoid*) or "avoidant disorder" or (phobi* near/2 neuros*) or "phobic disorder*" or (school* near/2 (anxiet* or anxious* or phobi* or refuse or refusal)) or shy or shyness or "specific phobia*":kw

ADDITIONAL SEARCHES
Please see: Clark DM, Pilling S, Mayo-Wilson E, *et al.* (2013). Social anxiety disorder: The NICE guideline on the recognition, assessment and treatment of social anxiety disorder. Royal College of Psychiatrists: London. (http://www.nice.org.uk/Guidance/CG159/Documents)

APPENDIX 2: OUTCOME MEASURES INCLUDED

Continuous
Anxiety Disorders Interview Schedule (ADIS-IV): Fear and Avoidance[1]
Brief Social Phobia Scale[2,3]
Clinical Global Impression (CGI): Severity[4]
Fear of Negative Evaluation Scale[5]
Fear Questionnaire (FQ): Social Phobia[6]
Liebowitz Social Anxiety Scale[7]
Liebowitz Social Anxiety Scale –Self Report[8,9]
Social Avoidance and Distress Scale (SADS)[5]
Social Interaction Anxiety Scale (SIAS); Social Phobia Scale (SPS)[10]
Social Phobia Anxiety Inventory[11]

Dichotomous
Recovery (i.e. loss of diagnosis)

APPENDIX 3: CORRELATION AMONG MEASURES (CHANGE DURING TREATMENT)

Caption: Data from consecutive referrals to Centre for Anxiety Disorder and Trauma (CADAT) in Maudsley Hospital. All treated individuals received cognitive therapy. For individual correlations, *n* ranges from 365-437)

	SPAI-SP	SPS	SIAS	FNE	BDI	BAI
LSAS	.77	.64	.70	.64	.41	.40
SPAI-SP	-	.63	.70	.70	.36	.36
SPS		-	.54	.50	.41	.40
SIAS			-	.64	.39	.32
FNE				-	.29	.26
BDI					-	.52

ADIS: Anxiety Disorders Interview Schedule – Severity
SPAI-SP: Social Phobia Anxiety Inventory – Social Phobia Subscale
SPS: Social Phobia Scale
SIAS: Social Interaction Anxiety Scale
FNE: Fear of Negative Evaluation Scale
BDI: Beck Depression Inventory
BAI: Beck Anxiety Inventory

APPENDIX 4: CONTINUOUS EFFECTS COMPARED WITH OBSERVED RECOVERY

Caption: The log-odds ratio (LOR) of recovery compared with the implied LOR from the standardised mean difference (SMD).[12] We assumed a linear relationship between continuous outcomes and recovery, forcing the intercept at zero. The result suggests that continuous measures may underestimate loss of diagnosis (slope=1.15, 95% CrI (0.97, 1.36)). This correspondence, and its uncertainty, was used to estimate the SMD from studies reporting only recovery and vice-versa. Studies with large effects, and studies with large difference between the effect based on dichotomous or continuous data are named.

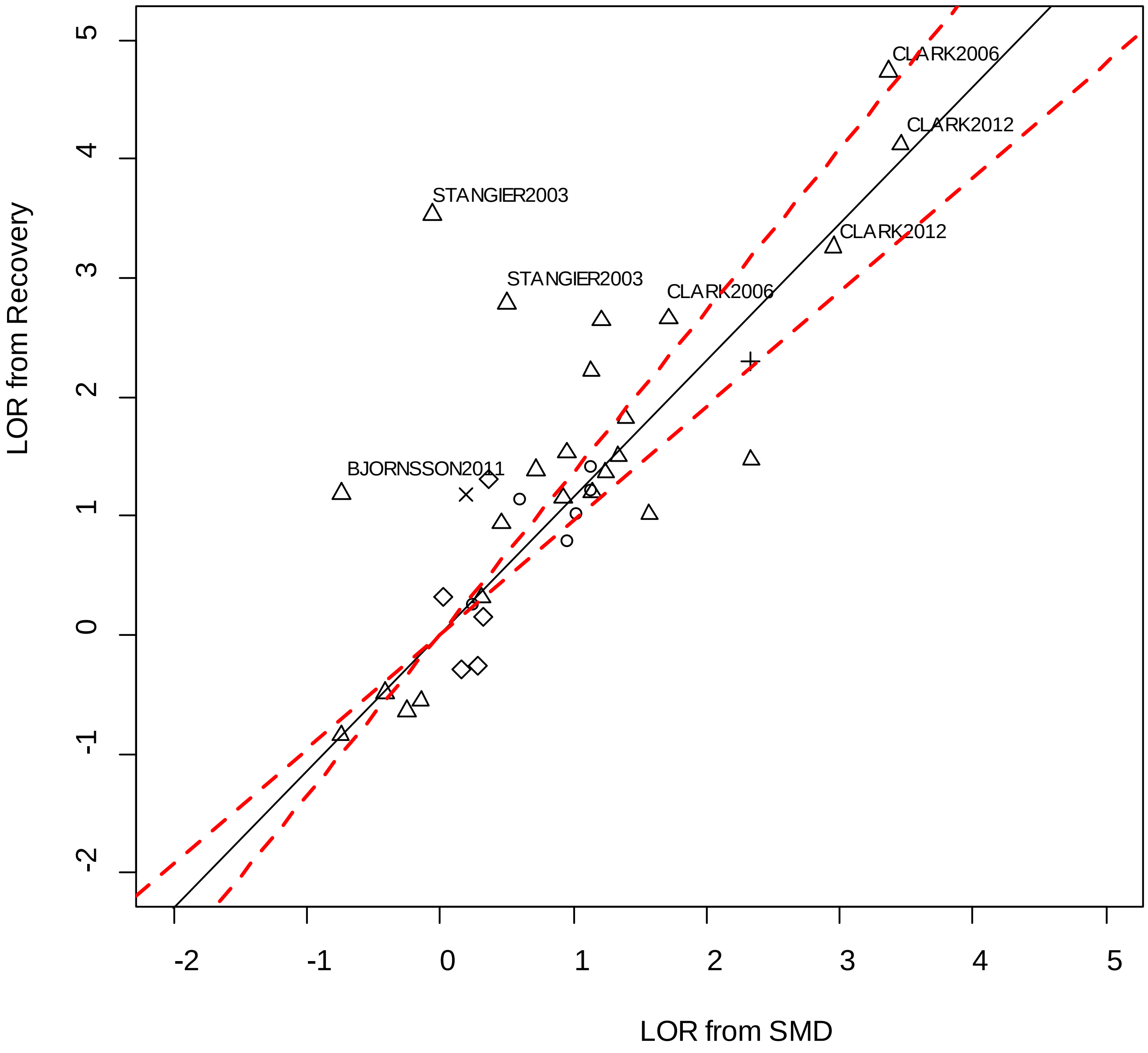

APPENDIX 5: WINBUGS CODE

I. Priors

The between-trials standard deviation was given a Uniform (0,5) prior.

The within-class mean treatment effects were given vague priors $m_j \sim N(0,100^2)$ and the within-class variability parameters had priors $1/\tau_j^2 \sim Gamma(a,b)$ with *a*=3.9 and *b*=0.35 chosen so that the mean of the within class standard deviation is the same as the posterior mean of the between-trial standard deviation (estimated in a previous run of the model without class effects) and the Credible interval can go from approximately half to double that mean.
For treatments belonging to a class formed only of themselves, the relative treatment effects were given non-informative priors $d_{1,k} \sim N(0,100^2)$.

II. WinBUGS code

```
# Normal likelihood, identity link for trial-level data given as SMD
# Class model: Random effects model for multi-arm trials
# Includes calibration regression for Recovery
model{ # *** PROGRAM STARTS
# 2 ARM STUDIES SMD
for(i in 1:ns2) { # LOOP THROUGH 2-ARM STUDIES
 prec[i,2] <- 1/Var[i,2] # set precisions
 y[i,2] ~ dnorm(delta[i,2],prec[i,2]) # normal likelihood for 2-arm trials
#Deviance contribution for trial i
 resdev[i] <- (y[i,2]-delta[i,2])*(y[i,2]-delta[i,2])*prec[i,2]
 }
# 3 ARM STUDIES SMD
for(i in 1:ns3) { # LOOP THROUGH THREE-ARM STUDIES
 for (k in 1:(na[i+ns2]-1)) { # set variance-covariance matrix
 for (j in 1:(na[i+ns2]-1)) {
 Sigma[i,j,k] <- V[i+ns2]*(1-equals(j,k)) + Var[i+ns2,k+1]*equals(j,k)
 }
 }
# Precision matrix
 Omega[i,1:(na[i+ns2]-1),1:(na[i+ns2]-1)] <- inverse(Sigma[i,,])
# multivariate normal likelihood for 3-arm trials
 y[i+ns2,2:na[i+ns2]] ~ dmnorm(delta[i+ns2,2:na[i+ns2]],Omega[i,1:2,1:2])
# Deviance contribution for trial i
 for (k in 1:(na[i+ns2]-1)){ # multiply vector & matrix
 ydiff[i,k]<- y[i+ns2,(k+1)] - delta[i+ns2,(k+1)]
 z[i,k]<- inprod2(Omega[i,k,1:2], ydiff[i,1:2])
              }
 resdev[i+ns2]<- inprod2(ydiff[i,1:(na[i+ns2]-1)], z[i,1:(na[i+ns2]-1)])
 }
# 4 ARM STUDIES SMD
for(i in 1:ns4) { # LOOP THROUGH FOUR-ARM STUDIES
 for (k in 1:(na[i+ns2+ns3]-1)) { # set variance-covariance matrix
 for (j in 1:(na[i+ns2+ns3]-1)) {
 Sigma4[i,j,k] <- V[i+ns2+ns3]*(1-equals(j,k)) + Var[i+ns2+ns3,k+1]*equals(j,k)
 }
 }
# Precision matrix
 Omega4[i,1:(na[i+ns2+ns3]-1),1:(na[i+ns2+ns3]-1)] <- inverse(Sigma4[i,,])
# multivariate normal likelihood for 4-arm trials
 y[i+ns2+ns3,2:na[i+ns2+ns3]] ~ dmnorm(delta[i+ns2+ns3,2:na[i+ns2+ns3]],Omega4[i,1:3,1:3])
#Deviance contribution for trial i
 for (k in 1:(na[i+ns2+ns3]-1)){ # multiply vector & matrix
 ydiff4[i,k]<- y[i+ns2+ns3,(k+1)] - delta[i+ns2+ns3,(k+1)]
 z4[i,k]<- inprod2(Omega4[i,k,1:3], ydiff4[i,1:(na[i+ns2+ns3]-1)])
              }
 resdev[i+ns2+ns3]<- inprod2(ydiff4[i,1:(na[i+ns2+ns3]-1)], z4[i,1:(na[i+ns2+ns3]-1)])
 }
# 5 ARM STUDIES SMD
for(i in 1:ns5) { # LOOP THROUGH FIVE-ARM STUDIES
# set variance-covariance matrix
 for (k in 1:(na[i+ns-ns5]-1)) { # set variance-covariance matrix
 for (j in 1:(na[i+ns-ns5]-1)) {
 Sigma5[i,j,k] <- V[i+ns-ns5]*(1-equals(j,k)) + Var[i+ns-ns5,k+1]*equals(j,k)
 }
 }
# Precision matrix
 Omega5[i,1:(na[i+ns-ns5]-1),1:(na[i+ns-ns5]-1)] <- inverse(Sigma5[i,,])
# multivariate normal likelihood for 5-arm trials
 y[i+ns-ns5,2:na[i+ns-ns5]] ~ dmnorm(delta[i+ns-ns5,2:na[i+ns-ns5]],Omega5[i,1:4,1:4])
# Deviance contribution for trial i
 for (k in 1:(na[i+ns-ns5]-1)){ # multiply vector & matrix
 ydiff5[i,k]<- y[i+ns-ns5,(k+1)] - delta[i+ns-ns5,(k+1)]
 z5[i,k]<- inprod2(Omega5[i,k,1:4], ydiff5[i,1:(na[i+ns-ns5]-1)])
              }
 resdev[i+ns-ns5]<- inprod2(ydiff5[i,1:(na[i+ns-ns5]-1)], z5[i,1:(na[i+ns-ns5]-1)])
 }
# ALL STUDIES WITH SMD, RE MODEL
for(i in 1:ns){ # LOOP THROUGH ALL STUDIES
 w[i,1] <- 0 # adjustment for multi-arm trials is zero for control arm
```

```
 delta[i,1] <- 0 # treatment effect is zero for control arm
 for (k in 2:na[i]) { # LOOP THROUGH ARMS
# trial-specific LOR distributions
 delta[i,k] ~ dnorm(md[i,k],taud[i,k])
# mean of random effects distributions, with multi-arm trial correction
 md[i,k] <- d[t[i,k]] - d[t[i,1]] + sw[i,k]
# precision of random effects distributions (with multi-arm trial correction)
 taud[i,k] <- tau *2*(k-1)/k
# adjustment, multi-arm RCTs
 w[i,k] <- (delta[i,k] - d[t[i,k]] + d[t[i,1]])
# cumulative adjustment for multi-arm trials
 sw[i,k] <- sum(w[i,1:k-1])/(k-1)
 }
 }
totresdev <- sum(resdev[]) # Total Residual Deviance
#
d[1] <- 0 # treatment effect (SMD) is zero for reference treatment
# own class treatments with zero variance
d[2] ~ dnorm(0, .0001) # vague prior for treatment effects (SMD)
d[3] ~ dnorm(0, .0001) # vague prior for treatment effects (SMD)
d[29] ~ dnorm(0, .0001) # vague prior for treatment effects (SMD)
# own class treatments borrowing variance
d[4] ~ dnorm(m[D[4]], prec2[5]) # variance from class E=5
d[12] ~ dnorm(m[D[12]], prec2[9]) # variance from class I=9
# Class model (informative prior for within-class precision)
for (k in 5:11){
 d[k] ~ dnorm(m[D[k]], prec2[D[k]]) # treatment effects from Class (SMD)
 }
for (k in 13:28){
 d[k] ~ dnorm(m[D[k]], prec2[D[k]]) # treatment effects from Class (SMD)
 }
for (k in 30:nt){
 d[k] ~ dnorm(m[D[k]], prec2[D[k]]) # treatment effects from Class (SMD)
 }
m[1] <- 0
m[2] <- d[2] # no class effect
m[3] <- d[3] # no class effect
m[14] <- d[29] # no class effect
for (k in 4:13){
 m[k] ~ dnorm(0, .0001) # prior for mean class effect
 prec2[k] ~ dgamma(a,b) # prior for class precision
 sd2[k] <- pow(prec2[k], -0.5)
}
for (k in 15:nc){
 m[k] ~ dnorm(0, .0001) # prior for mean class effect
 prec2[k] ~ dgamma(a,b) # prior for class precision
 sd2[k] <- pow(prec2[k], -0.5)
}
#
sd ~ dunif(0,5) # vague prior for between-trial SD
tau <- pow(sd,-2) # between-trial precision = (1/between-trial variance)
#
# REGRESSION FOR RECOVERY
for (j in 1:nR){ # LOOP THROUGH STUDIES REPORTING RECOVERY
 lambda[j,1] <- 0 # treatment effect is zero for control arm
 mu[j] ~ dnorm(0,.0001) # vague priors for all trial baselines
 for (k in 1:naR[j]) { # LOOP THROUGH ARMS
 r[j,k] ~ dbin(p[j,k],n[j,k]) # binomial likelihood for recovery data
 logit(p[j,k]) <- mu[j] + lambda[j,k] # model for linear predictor
 rhat[j,k] <- p[j,k] * n[j,k] # expected value of the numerators
#Deviance contribution
 devR[j,k] <- 2 * (r[j,k] * (log(r[j,k])-log(rhat[j,k]))
 + (n[j,k]-r[j,k]) * (log(n[j,k]-r[j,k]) - log(n[j,k]-rhat[j,k]))) }
 for (k in 2:naR[j]) { # LOOP THROUGH ARMS
# regression equation: LOR(Rec) = beta * LOR(SMD)
 lambda[j,k] <- beta * (-3.1416/sqrt(3)) * delta[C[j],k]
 }
# summed residual deviance contribution for this trial
 resdevR[j] <- sum(devR[j,1:naR[j]])
}
totresdevR <- sum(resdevR[]) #Total Residual Deviance Recovery
```

```
beta ~ dnorm(0,.0001)
# get confidence bound for beta for plotting
for (j in 1:1001){
 X[j] <- -1 + (j-1)*0.005
 Y[j] <- beta * X[j]
 }
#
for (k in 1:nc) {
 mCal[k] <- (-3.1416/sqrt(3)) * beta * m[k]
# rank classes
 rkClass[k] <- rank(m[],k) # Smallest is best (i.e. rank 1)
 bestClass[k] <- equals(rkClass[k],1)
 }
for (k in 1:nt) {
 g[k] <- (-3.1416/sqrt(3)) * d[k] # Transform SMD to LOR (change sign)
 gCal[k] <- beta * g[k] # Transform SMD to LOR (change sign): Calibrated
# rank all treatments on SMD
 rk[k] <- rank(d[],k) # Smallest is best (i.e. rank 1)
 best[k] <- equals(rk[k],1)
 for (h in 1:nt) { prob[h,k] <- equals(rk[k],h) }
# rank all treatments on LOR (calibrated)
 rk.g[k] <- nt+1-rank(gCal[],k) # Largest is best (i.e. rank 1)
 best.g[k] <- equals(rk.g[k],1)
 for (h in 1:nt) { prob.g[h,k] <- equals(rk.g[k],h) }
 }
for (c in 1:(nt-1)) { # for each treatment
 for (k in (c+1):nt) {
 diff[c,k] <- d[k] - d[c] # pairwise SMDs
 lor[c,k] <- g[k] - g[c] # pairwise LORs
 or[c,k] <- exp(g[k] - g[c]) # Paiwise ORs
 lorCal[c,k] <- gCal[k] - gCal[c] # pairwise LORs (calibrated)
 orCal[c,k] <- exp(gCal[k] - gCal[c]) # Paiwise ORs (calibrated)
 rr[c,k] <- RR[k]/RR[c]
 }
 }
for (c in 1:(nc-1)) { # for each class
 for (k in (c+1):nc) {
 diffClass[c,k] <- m[k] - m[c] # class v class SMDs
 lorClass[c,k] <- mCal[k] - mCal[c] # class v class LORs (calibrated)
 orClass[c,k] <- exp(mCal[k] - mCal[c]) # class v class ORs (calibrated)
 rrClass[c,k] <- RRC[k]/RRC[c] # RR for class
 }
 }
# Provide estimates of treatment effects T[k] on the natural scale
# Given a Mean Effect, meanA, for 'standard' treatment A,
# with precision (1/variance) precA
A ~ dnorm(meanA,precA)
for (k in 1:nt) {
 logit(T[k]) <- A + gCal[k] # prob recovery for each treatment
 RR[k] <- T[k]/T[1] # relative risk for each treatment
 }
for (k in 1:nc) {
 logit(TClass[k]) <- A + mCal[k] # prob recovery for each class
 RRC[k] <- TClass[k]/TClass[1] # relative risk for each class
 }
} # *** PROGRAM ENDS
```

II. Sample WinBUGS data

```
# NOTE place multi-arm trials at the end: 3-arms, then 4-arms, then 5 arms
# 3 sets of data to load
# ns= number of studies reporting SMD;
# ns2,3,4,5= number of studies with 2,3,4 and 5 arms
# nt=number of treatments; nc = number of classes
# D = vector of treatment classes
# V is matrix with covariances for multi-arm trials
# nR = number of studies reporting Recovery (for calibration)
# C is vector of study ID for SMD <=> Recovery
# a, b = parameters of Gamma prior for within-class precision

list(ns=101, ns2=66, ns3=26, ns4=7, ns5=2, nt=41, nc=17,
D=c(1, 2, 3, 4, 5, 5, 6, 6, 7, 7, 7, 8, 9, 9, 9, 9, 9, 9, 9, 10, 10, 11, 11, 12, 12, 13, 13, 13, 14, 15, 15, 15, 16, 16, 16, 16, 17, 17, 17, 17, 17),
nR= 24, meanA=-2.629, precA=0.81, a=3.9, b=0.35)

# Recovery Data
naR[]   r[,1]   r[,2]   r[,3]   r[,4]   n[,1]   n[,2]   n[,3]   n[,4]   C[]   #   ID
2       12      46      NA      NA      102     102     NA      NA      3     #   1    ANDERSSON2012
2       0       17      NA      NA      21      31      NA      NA      4     #   2    BERGER2009
2       6       13      NA      NA      32      30      NA      NA      9     #   3    ANDERSSON2006
...
3       1       14      19      NA      27      28      27      NA      80    #   17   CLARK2012
3       5       6       12      NA      20      20      20      NA      82    #   18   CLARK2003
3       19      42      55      NA      146     144     144     NA      85    #   19   ALLGULANDER2004
3       6       9       19      NA      58      66      71      NA      90    #   20   RAPEE2009
3       15      16      13      NA      27      27      27      NA      91    #   21   BERGER2011
4       3       11      13      11      52      56      59      57      93    #   22   RAPEE2007
4       3       7       7       19      39      45      40      42      97    #   23   BLANCO2010
4       16      11      12      10      29      28      29      29      99    #   24   FURMARK2009b
END

# Symptoms of SA Data
na[]    t[,1]   t[,2]   t[,3]   t[,4]   t[,5]   y[,2]   Var[,2]   y[,3]   Var[,3]   y[,4]   Var[,4]   y[,5]   Var[,5]   V[]
        #               ID
2       1       6       NA      NA      NA      -0.916254782    0.101294737     NA      NA      NA      NA      NA
        NA      NA      #       1       BOWLER2012
2       1       7       NA      NA      NA      -0.956381011    0.151840971     NA      NA      NA      NA      NA
        NA      NA      #       2       ABRAMOWITZ2009
2       1       8       NA      NA      NA      -0.768207456    0.015673733     NA      NA      NA      NA      NA
        NA      NA      #       3       ANDERSSON2012
2       1       8       NA      NA      NA      -0.748858081    0.063047078     NA      NA      NA      NA      NA
        NA      NA      #       4       BERGER2009

...

3       1       5       7       NA      NA      -0.527617361    0.132720853     -0.58287419     0.122467826     NA
        NA      NA      NA      0.071428571     #       67      CHUNG2008
3       1       5       8       NA      NA      -0.667164493    0.039020537     -0.625564669    0.038909081     NA
        NA      NA      NA      0.025   #       68      FURMARK2009a
3       1       6       8       NA      NA      -0.41624354     0.051117555     -0.990528783    0.052775936     NA
        NA      NA      NA      0.028571429     #       69      TITOV2008c

...

4       3       24      24      24      NA      -0.494649276    0.151436384     0.069457394     0.143952076     -
0.33273846      0.144903181     NA      NA      0.076923077     #       98      SMITS2006
4       5       7       8       8       NA      0.136746976     0.053544459     0.080320801     0.052579209
        0.411722844     0.05291421      NA      NA      0.034482759     #       99      FURMARK2009b
5       2       15      15      15      18      -0.304880876    0.01006318      -0.569978222    0.010179183     -
0.386028735     0.010013582     -0.449313527    0.010003406     0.006060606     #       100     LADER2004
5       2       16      30      30      37      -0.671526072    0.026136869     -0.497003859    0.025200991     -
0.614339904     0.025542189     -0.531314484    0.025378379     0.017467249     #       101     DAVIDSON2004b
END
```

APPENDIX 6: CHARACTERISTICS OF EXCLUDED STUDIES, AND REFERENCES

STUDY ID	REASON FOR EXCLUSION
Alden 1989[13]	No usable data (paper reports means and SDs, but does not report group size for any outcomes).
Allsopp 1984[14]	No usable data (medication for mixed population; N=33 with agoraphobia or social phobia).
Amir 2009[15]	Not an eligible intervention (cognitive bias modification).
Amir 2012[16]	Not an eligible intervention (cognitive bias modification).
Atmaca 2002[17]	Population differed in severity from all included studies. Baseline LSAS aproximately 122 (rather than aproximately 78).
Barnett 2002[18]	Not an eligible intervention (olanzapine).
Beard 2011[19]	Not an eligible intervention (cognitive bias modification).
Boettcher 2011[20]	Not an eligible intervention (cognitive bias modification).
Botella 2010[21]	Not an eligible intervention (intervention for public speaking anxiety only).
Butler 1984[22]	No usable data (outcomes not reported in a format that can be extracted for meta-analysis).
Carlbring 2012[23]	Not an eligible intervention (cognitive bias modification).
Carlbring ongoing[24]	Ongoing study of internet-based CBT compared with attention training.
Cassin 2011[25]	Not an eligible intervention (not a bona fide therapy, outcomes measured immediately after intervention).
Clark 1991[26]	Not an eligible intervention (buspirone).
Clark ongoing[27]	Ongoing study of internet-based cognitive therapy, face-to-face cognitive therapy, and waitlist.
Craske 2011[28]	Interventions not connected (preference-based treatment versus treatment-as-usual).
Davidson 2002[29]	Could not locate full report. Unclear if this is related to a RCT of venlafaxine.
D'El Rey 2007[30]	The reported data are implausible (significant at the level p=0.000002 with 30 particiapnts) and could not be verified.
De Oliviera 2012[31]	Interventions not connected (two unique forms of psychotherapy).
Donahue 2009[32]	Not an eligible intervention (single dose of quetiapine).
Dogaheh 2011[33]	No usable data (study compares group and individual CBT, but only the Brief Fear of Negative Evaluation Scale is reported as a measure of social anxiety).
Fahlen 1995[34]	Not an eligible intervention (brofaromine).
Furmark 2005[35]	Not an eligible intervention (NK1A).
Emmelkamp 1985[36]	No usable data (paper reports means but not SDs; we did not impute SDs to connect Rational Emotive Therapy and Self-Instructional Training to the network).
Hayes 2006[37]	No usable data (intervention for social anxiety and comorbid alcohol misuse; no social anxiety outcomes reported).
Heeren 2012[38]	Not an eligible intervention (cognitive bias modification).

Heideman 2008[39]	No usable data (intervention for social anxiety and comorbid alcohol misuse; no social anxiety outcomes reported).
Hofmann 2004[40]	No usable data (compares two types of group therapy with waitlist; paper reports mediation analyses, but not main effects for each intervention).
Johnston 2011[41]	No usable data (outcomes collapsed across social anxiety and other diagnostic groups).
Katzenick 1995[42]	No usable data (outcomes reported only after crossover).
Kobak 2005[43]	Not an eligible intervention (St John's Wort).
Krylov 1996[44]	Could not locate full report (compares alprazolam and buspirone).
Lee 1997[45]	Could not locate full report (compares alprazolam and buspirone).
Lott 1997[46]	Not an eligible intervention (short-term study of a beta-blocker).
Marks 2004[47]	No usable data (computerised therapy for mixed population; disaggregated data not available).
Mersch 1995[48]	No usable data (waitlist group were assigned after the first phase of the study; data from two treatments merged for comparison with waitlist).
Mortberg 2006[49]	No usable data (reports F-tests only for significant interactions, so these were not extracted).
Muehlbacher 2005[50]	The reported data are implausible. The journal and the authors were unable to verify details upon request.
Mulkens 2001[51]	Not an eligible intervention (intervention for fear of blushing only).
Neubauer 2013[52]	Not an eligible intervention (cognitive bias modification).
Norton 2012[53]	No usable data. Although primary diagnosis was not related to improvement with a transdiagnostic intervention (n=12) or a diagnosis-specific intervention (n=13), change on the measure of social anxiety non-significantly favoured the specific intervention.
Norwegian University of Science and Technology 2011[54]	Could not locate full report (compares paroxetine, cognitive therapy, paroxetine with cognitive therapy, and pill placebo).
Noyes 1997[55]	No usable data (includes figures, but raw scores not given).
Ontiveros 2008[56]	Assigned in groups of 10, but method of assignment not reported and not clear from personal correspondence (unclear if random).
Peng 2003[57]	Could not locate full report. Unclear if this is a RCT.
Pollack 2013[58]	Not an eligible intervention (second-line treatment for sertraline non-responders).
Randall 2001[59]	No usable data (paroxetine for social anxiety and comorbid alcohol misuse; no social anxiety outcomes reported).
Ravindran 2009[60]	Not an eligible intervention (atamoxetine).
Schmidt 2009[61]	Not an eligible intervention (cognitive bias modification).
Schneider 2005[62]	No usable data (computerised therapy for mixed population; disaggregated data not available for 24 participants with primary social anxiety disorder).
Shaw 1979[63]	No usable data (desensitization, flooding, and social skills training; 30 participants with social phoabia).
Solyom 1981[64]	No usable data (medication for mixed population; disaggregated data not available for 40 participants with agoraphobia or social phobia).
Taylor 1997[65]	Not an eligible intervention (compared exposure with or without cognitive restructuring, but explicitly *not* as restructuring is used in clinical practice).

Tejedor 1996[66]	Could not locate full report (rational emotive therapy, behavioural procedures, and waitlist).
Taylor 2010[67]	Not an eligible intervention (compared two forms of exposure in a laboratory-based interaction to explore mediators of change).
Turner 1994[68]	Not an eligible intervention (short-term study of a beta-blocker).
Tyrer 1973[69]	No usable data (medication for mixed population; disaggregated data not available for 15 particaipnts with social anxiety disorder).
Vaishnavi 2007[70]	Not an eligible intervention (quetiapine).
Van Ameringen 2007[71]	Not an eligible intervention (nefazodone).
Van Ameringen ongoing[72]	Ongoing study of sertraline and placebo.
Van-Vliet 1992[73]	Not an eligible intervention (brofaromine).
Van-Vliet 1997[74]	Not an eligible intervention (buspirone).
Versiani 1997[75]	Not an eligible intervention (bromazepam).
Yang 1999[76]	Could not locate full report (compares systematic desensitization with or without therapist accompaniment).
Yoshinaga ongoing[77]	Ongoing study of CBT for sertraline non-responders.
Zhang 2000[78]	Not an eligible intervention (Taoist cognitive psychotherapy).
Zhang 2003[79]	Could not locate full report (compares paroxetine and alprazolam).

APPENDIX 7: CHARACTERISTICS OF INCLUDED STUDIES, AND REFERENCES

Study	Country	Funding	Severity	Age	Sex	Med	Rand	Post	Interventions	Unpublished data
Abramowitz 2009[80]	USA	Mayo Clinic and Mayo Foundation	NR; 56/NR	43	76	57	21	21	Self-help with support (book)<br>Waitlist	Methods (Unable to provide outcomes)
Alden 2011[81]	CAN	Social Sciences and Humanities Council of Canada	NR; 54/31	34	41	38	56	50	Group CBT<br>Waitlist	NA
Allgulander 1999[82-84]	SWE	Novo Nodisk	75; NR	39	NR	0	96	92	Paroxetine<br>Pill placebo	Outcomes
Allgulander 2004[85]	Many	Wyeth	85; NR	39	53	NR	436	389	Venlafaxine<br>Paroxetine<br>Pill placebo	Outcomes
Andersson 2006[86]	SWE	Swedish Research Council; Soderstrom-Konigska Foundation	68; 45/34	37	52	22	64	62	Exposure in vivo<br>Waitlist	NA
Andersson 2012[87]	SWE	Swedish Research Council	67; 49/38	38	60	14	204	204	Self-help with support (internet)<br>Waitlist	NA
Andrews 2011[88]	AUS	NA	NR; 56/43	32	41	NR	37	25	Group CBT<br>Self-help with support (internet)	NA
Asakura 2007[89]	JPN	Solvay; Meiji Seika Kaisha	88; NR	39	32	NR	273	265	Fluvoxamine<br>Pill placebo	Methods and outcomes
Baldwin 1999[90-92]	Many	SmithKline Beecham	87	36	54	0	290	290	Paroxetine<br>Pill placebo	Study summary
Berger 2009[93]	SWZ	Swiss National Science Foundation	71; 45/35	29	56	NR	52	52	Self-help with support (internet)<br>Waitlist	Methods
Berger 2011[94]	SWE; SWZ	Swiss National Science Foundation	83; 50/35	37	53	7	81	81	Self-help with support (internet)<br>Self-help without support (internet)	NA
Bjornsson 2011[95]	USA	NA	78; NR	20	47	0	45	45	Group CBT<br>Psychological placebo	NA
Blanco 2010[96]	USA	NIMH (USA)	74; 41/34	32	41	0	166	128	Group CBT (Heimberg) + Phenelzine<br>Group CBT (Heimberg)<br>Phenelzine<br>Pill placebo	NA
Blomhoff 2001[97]	NOR; SWE	Pfizer	NR; NR	40	60	NR	387	354	Sertraline<br>Pill placebo	NA
Borgeat 2009[98]	SWZ	NA	77;	40	53	NR	30	27	Group CBT	NA

			NR						Exposure in vivo	
Bowler 2012[99]	GBR	Wellcome Trust; Medial Research Council	NR	23	68	NR	63	42	Self-help with support (internet)<br>Waitlist	NA
Burrows 1997[100]	Many	Hoffmann-La Roche	81;<br>NR	36	43	0	NR	578	Moclobemide<br>Pill placebo	NA
Carlbring 2007[101-104]	Sweden	Swedish Research Council; Soderstrom-Konigska Foundation	70;<br>42/37	33	62	NR	60	57	Self-help with support (internet)<br>Waitlist	Methods
Chung 2008[105]	KOR	Korea Science and Engineering Foundation	NR;<br>53/46	26	40	NR	45	38	Self-help with support (book)<br>Self-help without support (book)<br>Waitlist	NA
Clark 2003[106, 107]	GBR	Wellcome Trust	75;<br>45/33	33	52	0	60	60	Cognitive therapy<br>Fluoxetine<br>Pill placebo	NA
Clark 2006[108, 109]	GBR	Wellcome Trust	77;<br>47/32	32	44	28	62	62	Cognitive therapy<br>Exposure in vivo<br>Waitlist	NA
Clark 2012[110]	GBR	NA	82;<br>51/38	33	NR	28	82	82	Cognitive therapy<br>Waitlist	Methods and outcomes
Cottraux 2000[111]	FRA	French Ministry of Health	77;<br>NR	34	59	0	57	55	Individual CBT<br>Supportive therapy	NA
Davidson 1993[112, 113]	USA	NA	78;<br>NR	37	43	0	75	70	Clonazepam<br>Pill placebo	NA
Davidson 2004a[114-116]	USA	Solvay	90;<br>NR	37	36	0	279	247	Fluvoxamine<br>Pill placebo	Methods and outcomes
Davidson 2004b[117, 118]	USA	NIMH (USA)	80;<br>NR	37	46	0	295	295	Group CBT + Fluoxetine<br>Group CBT<br>Fluoxetine<br>Pill placebo	NA
Emmelkamp 2006[119]	NED	GGz Groningen; Mental Health Research Foundation	NR;<br>NR	34	52	NR	62	56	Individual CBT<br>Psychodynamic psychotherapy<br>Waitlist	NA
Feltner 2011[120]	USA	Pfizer	90;<br>NR	35	41	0	329	164	Pregabalin<br>Pill placebo	NA
Furmark 2002[121]	SWE	Swedish Council for Research in Humanities and Social Sciences;	NR;<br>34/29	35	44	0	18	18	Group CBT<br>Citalopram<br>Waitlist	Methods and outcomes

		Bank of Sweden Tercentenary Foundation; Swedish Brain Foundation								
Furmark 2005[122]	SWE	GlaxoSmithKline	70; 49/34	32	53	NR	36	36	Citalopram<br>Pill placebo	Methods and outcomes
Furmark 2009a[104, 123, 124]	SWE	Swedish Research Council. Authors receive royalties.	70; 49/37	36	68	67	120	120	Self-help with support (internet)<br>Self-help without support (book)<br>Waitlist	NA
Furmark 2009b[104, 123, 124]	SWE	Swedish Research Council. Authors receive royalties.	73; 50/39	35	68	14	115	115	Self-help with support (internet)<br>Self-help with support (book)<br>Self-help without support (book)	NA
Gelernter 1991[125, 126]	USA	NIMH (USA)	NR; NR	37	63	0	65	57	Group CBT (Heimberg)<br>Phenelzine<br>Alprazolam<br>Pill placebo	NA
Goldin 2012[127]	USA	NIMH (USA)	83; NR	33	46	0	75	75	Individual CBT (Hope)<br>Waitlist	Methods
Gruber 2001[128]	GER; USA	Deutscher Akademischer Austauschdienst (German Academic Exchange Service)	NR; NR/30	42	52	17	54	46	Group CBT (Heimberg)<br>Waitlist	NA
GSK 2006[129]	JPN	GlaxoSmithKline	81; NR	37	52	NR	400	395	Paroxetine<br>Paroxetine<br>Pill placebo	Study summary
Hedman 2011[124, 130, 131]	Sweden	Stockholm County Council; Bror Gadelius Fund	70; 48/33	35	36	25	126	126	Group CBT (Heimberg)<br>Self-help with support (internet)	NA
Heimberg 1990[132, 133]	USA	NIMH (USA)	NR	30	45	NR	49	40	Group CBT (Heimberg)<br>Psychological placebo	Methods
Heimberg 1998[134-136]	USA	NIMH (USA)	60; 45/29	35	50	0	133	107	Group CBT (Heimberg)<br>Phenelzine<br>Pill placebo<br>Psychological placebo	Methods and outcomes
Herbert 2004[137]	USA	NIMH (USA)	NR	34	44	NR	34	34	Individual CBT<br>Individual CBT	NA
Herbert 2005[138]	USA	NIMH (USA)	NR	34	57	23	65	65	Group CBT (Heimberg)	NA

Hope 1995[139]	USA	Internal funding	NR	34	50	30	40	33	Group CBT (Heimberg)<br>Exposure in vivo<br>Waitlist	NA
Jazaieri 2012[140]	USA	NIMH (USA)	87;<br>45/NR	34	52	0	56	42	Mindfulness stress reduction<br>Exercise promotion	NA
Kasper 2005[141, 142]	Many	Lundbeck	95;<br>NR	38	45	0	358	353	Escitalopram<br>Pill placebo	Methods and outcomes
Knijnik 2004[143]	BRA	NA	85;<br>NR	32	30	NR	40	30	Psychodynamic psychotherapy<br>Psychological placebo	NA
Knijnik 2008[144]	BRA	Roche; NIH	92;<br>NR	33	61	0	59	57	Psychodynamic + Clonazepam<br>Clonazepam	NA
Kobak 2002[145]	USA	Eli Lilly	82;<br>NR	39	58	NR	60	60	Fluoxetine<br>Pill placebo	Study summary
Koszycki 2007[146]	CAN	Internal funding	NR;<br>45/34	38	53	28	53	53	Group CBT (Heimberg)<br>Mindfulness stress reduction	NA
Lader 2004[147, 148]	Many	Lundbeck	94;<br>NR/NR	37	53	0	839	820	Paroxetine<br>Escitalopram<br>Pill placebo	Methods and outcomes
Ledley 2009[149]	USA	NA	68;<br>43/33	35	58	26	38	34	Individual CBT (Hope)<br>Waitlist	NA
Leichsenring 2013[150]	GER	German Federal Ministry of Education and Research	72;<br>NR	35	55	0	514	495	C&W CT shortened sessions<br>Psychodynamic psychotherapy<br>Waitlist	Outcomes
Lepola 2004[151]	Many	GlaxoSmithKline	78;<br>NR	39	50	0	375	370	Paroxetine<br>Pill placebo	Study summary
Liebowitz 1990[152-154]	USA	NIMH (USA)	NR;<br>NR	34	31	0	85	74	Phenelzine<br>Atenolol<br>Pill placebo	NA
Liebowitz 2002[155-157]	CAN; USA	SmithKline Beecham	77;<br>NR	37	41	NR	384	360	Paroxetine<br>Paroxetine<br>Pill placebo	Study summary
Liebowitz 2003[157-159]	USA	Pfizer	93;<br>NR	35	40	0	415	401	Sertraline<br>Pill placebo	NA
Liebowitz 2005a[160, 161]	USA	Wyeth	89;<br>NR	35	45	NR	280	271	Venlafaxine<br>Pill placebo	NA
Liebowitz 2005b[162]	USA	Wyeth	86;<br>NR	36	46	0	440	413	Venlafaxine<br>Paroxetine	NA

									Pill placebo	
Lipsitz 2008[163]	USA	NIMH (USA)	66;<br>44/29	35	43	NR	70	52	Interpersonal psychotherapy<br>Supportive therapy	NA
Matick 1988[164]	AUS	Australian Government	NR	37	53	NR	51	44	Group CBT<br>Exposure in vivo	NA
Matick 1989[165]	AUS	Australian Government	NR;<br>35/37	42	53	NR	43	37	Group CBT<br>Exposure in vivo<br>Waitlist	NA
McEvoy 2009[166]	AUS	NA	NR;<br>57/38	31	37	56	75	60	Group CBT	NA
Morgan 1999[167]	AUS	NA	NR;<br>54/42	32	47	47	36	30	Group CBT	Methods and outcomes
Mortberg 2007[168–170]	SWE	Boethius Foundation; Soderstrom-Konigska Foundation; The Organon Foundation; Stockholm County Council	75;<br>48/37	35	63	NR	100	100	C&W CT shortened sessions<br>Group CBT	NA
Munjack 1990[171]	USA	Roche	97;<br>NR	33	75	0	23	20	Clonazepam<br>Waitlist	NA
Oosterbaan 2001[172]	NED	Hoffmann-La Roche	68;<br>NR	37	42	0	82	67	Individual CBT<br>Moclobemide<br>Pill placebo	NA
Otto 2000[173]	USA	Roche	68;<br>45/30	40	40	0	45	45	Group CBT (Heimberg)<br>Clonazepam	NA
Pande 1999[174]	USA	Parke-Davis (now Pfizer)	85;<br>NR	36	43	0	69	69	Gabapentin<br>Pill placebo	Methods
Pande 2004[175,176]	USA	Parke-Davis (now Pfizer)	79;<br>NR	38	42	0	135	135	Pregabalin<br>Pill placebo	Methods
Pfizer 2007[177]	Many	Pfizer	93;<br>NR	37	45	NR	374	296	Paroxetine<br>Pregabalin<br>Pill placebo	Study summary
Piet 2010[178]	DEN	NA	65;<br>46/35	22	69	0	26	26	Group CBT<br>Mindfulness cognitive therapy	NA
Prasko 2003[179-183]	CZE	MSMT CR 1M0517	93;<br>NR	28	52	NR	81	66	Individual CBT + Moclobemide<br>Individual CBT + Pill placebo<br>Moclobemide	NA
Rapee 2007[184]	AUS	NA	NR;	36	51	32	224	224	Group CBT	Methods

			54/37						Group CBT<br>Self-help without support (book)<br>Waitlist	
Rapee 2009[185]	AUS	National Health and Medical Research Council of Australia	NR;<br>54/35	34	52	27	195	183	Group Enhanced CBT<br>Group CBT<br>Psychological placebo	NA
Rickels 2004[186]	USA	NA	89;<br>NR	41	43	0	276	261	Venlafaxine<br>Pill placebo	NA
Robillard 2010[187]	CAN	NA	78;<br>NR/34	35	71	NR	45	45	Individual CBT<br>Waitlist	NA
Salaberria 1998[188]	ESP	Internal funding	NR	31	48	NR	71	56	Group CBT<br>Exposure in vivo<br>Waitlist	Methods
Schneier 1998[189,190]	USA	NIMH (USA)	79;<br>NR	35	40	0	77	77	Moclobemide<br>Pill placebo	NA
Schutters 2010[191]	NED	Organon	71;<br>NR	39	57	0	60	60	Mirtazapine<br>Pill placebo	Methods and outcomes
Seedat 2004[192]	USA	GlaxoSmithKline	95;<br>NR	31	21	0	28	28	Paroxetine + Clonazepam<br>Paroxetine	NA
Smits 2006[193]	USA	Society for a Science of Clinical Psychology	73;<br>NR	22	57	NR	77	68	Exposure in vivo<br>Psychological placebo	NA
Stangier 2003[194]	GER	NA	NR;<br>43/31	39	49	5	95	65	C&W CT shortened sessions<br>Group CBT<br>Waitlist	Outcomes
Stangier 2011[195]	GER	German Research Foundation; Wellcome Trust; NIHR	67;<br>NR	36	56	0	117	111	C&W CT shortened sessions<br>Interpersonal psychotherapy<br>Waitlist	NA
Stein 1998[92, 196]	CAN;<br>USA	SmithKline Beecham	81;<br>NR	36	53	0	187	182	Paroxetine<br>Pill placebo	Study summary
Stein 1999[197]	USA	Pharmacia; Upjohn	NR	39	36	0	92	86	Fluvoxamine<br>Pill placebo	Methods and outcomes
Stein 2002[198]	ZA	Hoffmann-La Roche	76;<br>NR	34	47	0	390	377	Moclobemide<br>Pill placebo	NA
Stein 2005[199]	USA	Wyeth	89;<br>NR	37	42	0	395	364	Venlafaxine<br>Pill placebo	NA
Stravynski 2000[200]	CAN	NA	NR	38	58	NR	68	60	Exposure in vivo<br>Social skills training	NA

Tauscher 2010[201]	USA	Eli Lilly	NR	NR	NR	NR	189	126	Paroxetine<br>Pill placebo	NA
Titov 2008a[202-204]	AUS	Internal funding	NR;<br>54/35	38	59	30	105	99	Self-help with support (internet)<br>Waitlist	Methods
Titov 2008b[202, 204, 205]	AUS	Internal funding	NR;<br>57/35	37	63	26	88	81	Self-help with support (internet)<br>Waitlist	Methods
Titov 2008c[204, 206]	AUS	NA	NR;<br>53/34	38	61	22	98	95	Self-help with support (internet)<br>Self-help without support (internet)<br>Waitlist	Methods
Titov 2009a[207]	AUS	NA	NR;<br>54/36	39	56	28	85	82	Self-help with support (internet)	NA
Titov 2009b[208]	AUS	NA	NR;<br>54/34	41	52	22	163	163	Self-help with support (internet)<br>Self-help without support (internet)	NA
Titov 2010[209]	AUS	NA	NR;<br>53/34	44	47	16	113	108	Self-help without support (internet)	NA
Van-Ameringen 2001[210-214]	CAN	Pfizer	NR	36	44	0	204	203	Sertraline<br>Pill placebo	NA
Van-Vliet 1994[215, 216]	NED	NA	70;<br>NR	35	57	0	30	28	Fluvoxamine<br>Pill placebo	NA
Versiani 1992[217]	BRA	NA	NR;<br>NR/66	NR	NR	0	78	78	Phenelzine<br>Moclobemide<br>Pill placebo	NA
Westenberg 2004[116, 218, 219]	Many	Solvay	95;<br>NR	38	52	0	300	294	Fluvoxamine<br>Pill placebo	NA
Willutzki 2004[220-222]	GER	German Research Foundation	NR;<br>45/36	38	44	NR	83	69	Individual CBT<br>Individual CBT	NA
Wong 2006[223]	CHI	Internal funding	74;<br>NR	35	41	100	34	34	Group CBT (Heimberg)<br>Waitlist	NA
Zhang 2005[224]	USA	UCB Pharma	88;<br>NR	38	53	0	19	16	Levetiracetam<br>Pill placebo	NA

Country: Countries from which participants were recruited.
Funding: External sources of funding acknowledged.
Severity: Baseline scores on the Liebowitz Social Anxiety Scale (LSAS); and the Social Interaction Anxiety Scale (SIAS)/ Social Phobia Scale (SPS)
Age: Mean years of age.
Sex: Percentage of participants enrolled who were female.
Med: Percentage of participants taking medication (e.g. SSRI or benzodiazepine) at baseline. Participants were normally on a stable dose and agreed not to change their dose during the trial.
Rand: Number of participants randomised.
Post: Number of participants who completed assessment at post-treatment.

Interventions: Name of all interventions assessed. Studies with more than one example of an intervention contributed to estimates of within-group variance.
Unpublished data: Type of unpublished data obtained from authors or manufacturers.
NA: Not applicable
NR: Not reported

APPENDIX 8: BASELINE CHARACTERISTICS BY COMPARISON

Caption: For each comparison, the number of trials and participants and baseline characteristics are provided.

Intervention 1	Intervention 2	Trials	Participants	Duration	Age	Sex	Med
PSYP	PILL	1	133	12	35	50	0
ACV	PILL	5	185	11	37	45	0
BZD	PILL	2	70	11	37	53	0
BZD	WAIT	1	23	8	33	75	0
MAOI	BZD	1	65	12	37	63	0
MAOI	PILL	9	135	11	35	45	0
MAOI	PSYP	1	133	12	35	50	0
NSSA	PILL	1	60	12	39	57	0
SSRI	ACV	1	374	11	37	45	NR
SSRI	PILL	27	287	14	37	48	0
SSRI	WAIT	1	18	9	35	44	0
EXPO	PSYP	1	77	1	22	57	NR
EXPO	WAIT	4	52	10	36	50	27
GCBT	BZD	2	55	12	39	52	0
GCBT	EXPO	5	47	8	37	51	30
GCBT	MAOI	3	121	12	35	51	0
GCBT	OTHR	2	40	8	30	61	14
GCBT	PILL	4	165	13	35	50	0
GCBT	PSYP	4	106	11	30	49	9
GCBT	SHNS	1	224	12	36	51	32
GCBT	SHWS	2	82	12	34	39	25
GCBT	SSRI	2	157	12	36	45	0
GCBT	WAIT	9	71	15	37	48	32
ICBT	GCBT	2	98	23	37	56	5
ICBT	EXPO	1	62	14	32	44	28
ICBT	MAOI	2	82	21	33	47	0
ICBT	OTHR	2	87	16	35	58	0
ICBT	OTHR	2	288	20	35	54	0
ICBT	PILL	2	71	16	35	47	0
ICBT	SSRI	1	60	16	33	52	0
ICBT	WAIT	9	121	20	35	8	12
OTHR	EXER	1	56	8	34	52	0
OTHR	WAIT	1	117	20	36	56	0
PDPT	PSYP	1	40	12	32	30	NR
PDPT	WAIT	2	288	20	35	54	0
SHNS	WAIT	5	106	12	41	55	46
SHWS	SHNS	6	104	9	36	57	26
SHWS	WAIT	9	88	9	35	61	36
COMB	BZD	1	59	12	33	61	0
COMB	GCBT	2	231	13	35	44	0
COMB	ICBT	1	81	26	28	52	NR
COMB	MAOI	2	124	19	30	47	0
COMB	PILL	2	231	13	35	44	0
COMB	SSRI	2	162	12	34	34	0

Duration: Length of treatment (in weeks); **Age**: Mean years of age; **Sex**: Percentage of participants enrolled who were female. **Med**: Percentage of participants taking medication (e.g. SSRI or benzodiazepine) at baseline.

APPENDIX 9: RISK OF BIAS FOR EACH STUDY

Caption: For each domain, studies were rated as having a high (-), low (+), or unclear (?) risk of bias using the Cochrane Risk of Bias tool.

	Random sequence generation (selection bias)	Allocation concealment (selection bias)	Blinding of participants and personnel	Blinding of outcome assessment (detection bias)	Incomplete outcome data (attrition bias)	Selective reporting (reporting bias)
Abramowitz 2009	+	+	-	+	+	?
Alden 2011	?	?	-	+	+	?
Allgulander 1999	+	+	+	+	-	+
Allgulander 2004	+	+	+	+	+	+
Andersson 2006	+	+	-	-	+	?
Andersson 2012	+	+	-	+	+	+
Andrews 2011	+	+	-	+	-	+
Asakura 2007	+	+	+	+	+	+
Baldwin 1999	+	+	+	+	+	?
Berger 2009	+	+	-	+	+	+
Berger 2011	+	+	-	-	+	?
Bjornsson 2011	?	?	-	+	-	?
Blanco 2010	+	+	-	+	-	?
Blomhoff 2001	+	+	-	?	+	+
Borgeat 2009	?	?	-	+	+	?
Bowler 2012	?	?	-	+	-	?
Burrows 1997	+	+	+	+	?	?
Carlbring 2007	+	+	-	+	+	?
Chung 2008	?	?	-	+	+	?
Clark 2003	+	+	-	+	+	+
Clark 2006	+	+	-	+	+	+
Clark 2012	+	+	-	+	+	+
Cottraux 2000	+	+	-	+	+	?
Davidson 1993	+	+	+	+	+	?
Davidson 2004a	+	+	+	+	+	+
Davidson 2004b	+	+	-	+	-	-
Emmelkamp 2006	?	?	-	+	+	?
Feltner 2011	+	+	+	+	-	?
Furmark 2002	+	?	-	-	+	-
Furmark 2005	+	+	+	+	+	-

Furmark 2009a	+	+	-	+	+	+
Furmark 2009b	+	+	-	+	+	+
Gelernter 1991	?	?	-	+	+	-
Goldin 2012	+	+	-	+	+	+
Gruber 2001	?	?	-	+	-	?
GSK 2006	+	+	+	+	+	+
Hedman 2011	+	+	-	+	+	+
Heimberg 1990	+	?	-	+	+	+
Heimberg 1998	+	+	-	+	+	+
Herbert 2004	?	?	-	+	-	?
Herbert 2005	?	?	-	+	+	?
Hope 1995	?	?	-	+	+	?
Jazaieri 2012	+	+	-	+	-	?
Kasper 2005	+	+	+	+	+	+
Knijnik 2004	?	?	-	+	+	?
Knijnik 2008	+	+	-	+	+	?
Kobak 2002	+	+	+	+	+	+
Koszycki 2007	+	+	-	+	+	?
Lader 2004	+	+	+	+	+	+
Ledley 2009	?	?	-	+	+	?
Leichsenring 2013	+	+	-	+	-	-
Lepola 2004	+	+	+	+	+	+
Liebowitz 1990	+	?	+	+	-	?
Liebowitz 2002	+	+	+	+	+	+
Liebowitz 2003	+	+	+	+	+	?
Liebowitz 2005a	?	+	+	+	+	?
Liebowitz 2005b	+	+	+	+	+	-
Lipsitz 2008	+	+	-	+	-	+
Matick 1988	?	?	-	+	+	?
Matick 1989	?	?	-	+	+	?
McEvoy 2009	+	-	-	+	+	?
Morgan 1999	?	?	-	+	-	+
Mortberg 2007	+	+	-	+	+	?
Munjack 1990	+	-	-	+	-	?
Oosterbaan 2001	?	?	-	+	?	-
Otto 2000	?	?	-	+	+	?
Pande 1999	+	+	+	+	+	?
Pande 2004	+	+	+	+	-	?
Pfizer 2007	+	+	+	+	-	+
Piet 2010	+	+	-	-	+	?
Prasko 2003	?	?	-	+	+	?
Rapee 2007	+	+	-	?	+	?
Rapee 2009	+	+	-	+	+	?
Rickels 2004	+	+	+	+	+	+

Robillard 2010	?	?	-	+	?	?
Salaberria 1998	?	?	-	+	+	-
Schneier 1998	+	+	+	+	+	?
Schutters 2010	+	+	+	+	+	?
Seedat 2004	+	+	+	+	+	?
Smits 2006	?	?	-	+	-	?
Stangier 2003	+	-	-	+	-	+
Stangier 2011	+	+	-	+	+	?
Stein 1998	+	+	+	+	+	+
Stein 1999	+	+	+	+	-	+
Stein 2002	+	+	+	+	+	+
Stein 2005	+	+	+	+	-	-
Stravynski 2000	?	?	-	-	+	?
Tauscher 2010	?	?	+	+	-	-
Titov 2008a	+	+	-	+	+	+
Titov 2008b	+	+	-	+	+	+
Titov 2008c	+	+	-	+	+	+
Titov 2009a	+	+	-	+	+	+
Titov 2009b	+	+	-	+	+	+
Titov 2010	+	+	-	+	+	+
Van-Ameringen 2001	+	+	+	+	+	?
van-Vliet 1994	?	?	+	+	?	?
Versiani 1992	+	+	+	+	-	?
Westenberg 2004	+	+	+	+	+	?
Willutzki 2004	?	?	-	+	-	?
Wong 2006	+	+	-	+	-	?
Zhang 2005	+	+	+	+	-	?

APPENDIX 10: RISK OF BIAS SUMMARY

Caption: Risk of bias summary using the Cochrane Risk of Bias Tool for all interventions in the network analysis.

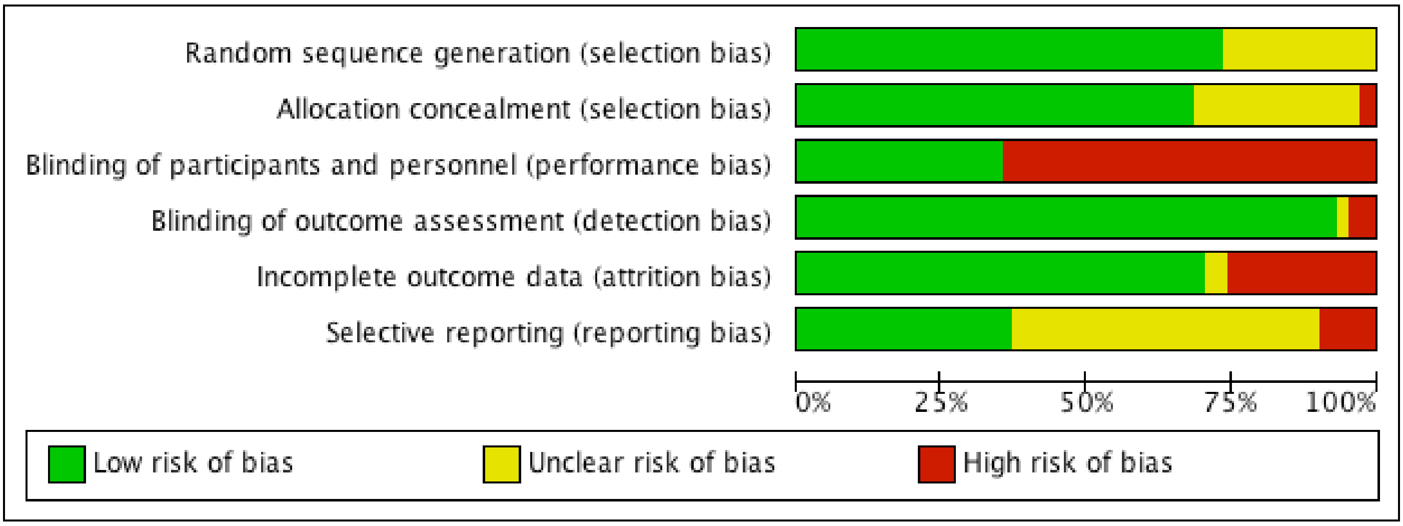

APPENDIX 11: NETWORK OF SPECIFIC TREATMENTS, CONTINUOUS OUTCOMES

Caption: Network diagram representing direct comparisons of individual interventions using all available data, which are arranged by class. The width of lines represents the number of trials making each direct comparison.

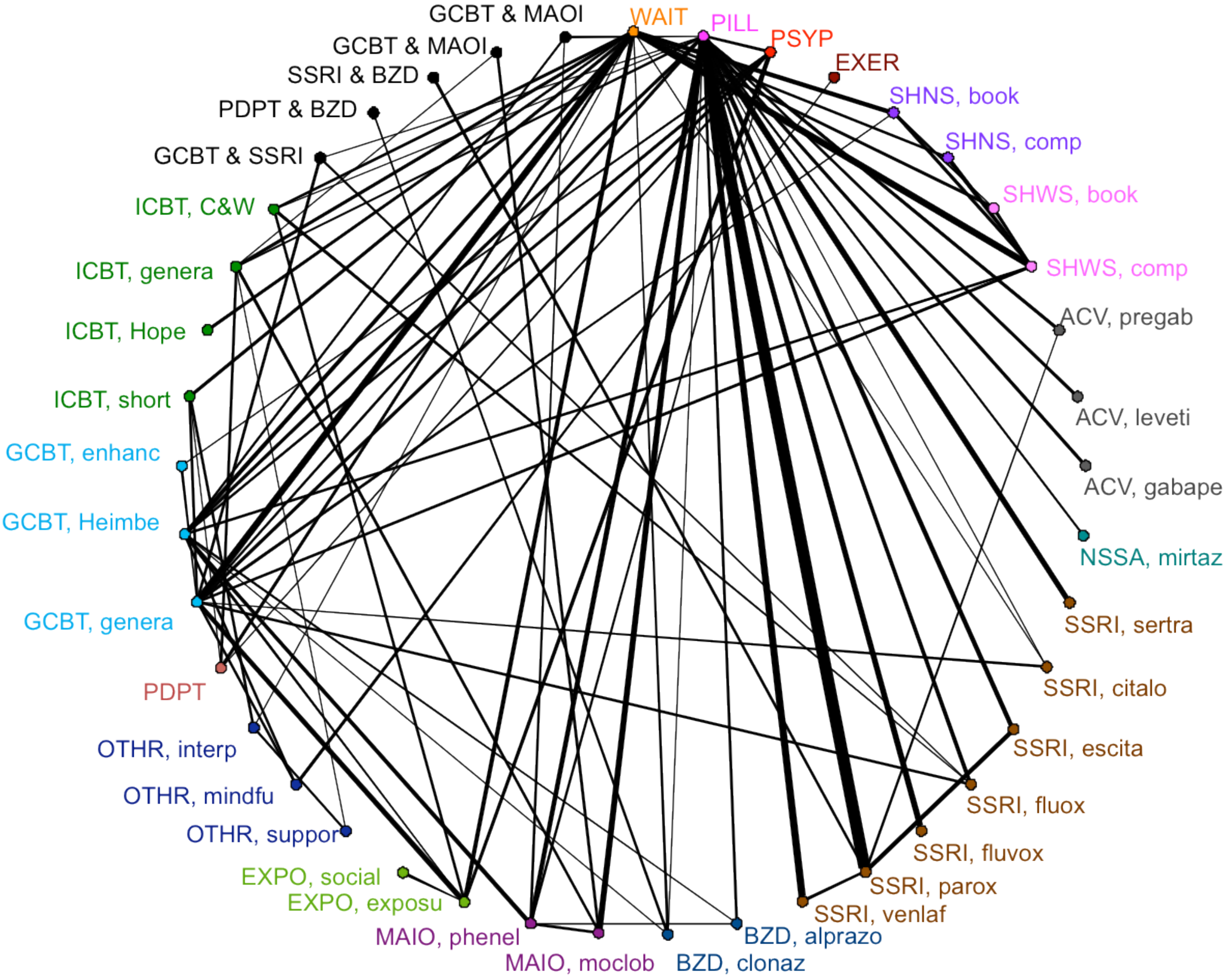

Waitlist (WAIT)
Waitlist
Pill placebo (PILL)
Pill placebo
Psychological placebo (PSYP)
Psychological placebo
Exercise promotion (EXER)
Exercise promotion
Self-help without support (SHNS)
Self-help book no support (SHNS, book)
Self-help internet no support (SHNS, comp)
Self-help with support (SHWS)
Self-help book w/ support (SHWS, book)
Self-help internet w/ support (SHWS, comp)
Anticonvulsants (ACV)
Pregabalin (ACV, pregab)
Levetiracetam (ACV, leveti)
Gabapentin (ACV, gabape)
Noradrenergic and specific serotonergic antidepressants (NSSA)
Mirtazapine (NSSA, mirtaz)
SSRIs & SNRIs (SSRI)
Sertraline (SSRI, sertra)
Citalopram (SSRI, citalo)
Escitalopram (SSRI, escita)
Fluoxetine (SSRI, fluox)
Fluvoxamine (SSRI, fluvox)
Paroxetine (SSRI, parox)
Venlafaxine (SSRI, venlaf)
Benzodiazepines (BZD)
Alprazolam (BZD, alprazo)
Clonazepam (BZD, clonaz)
Monoamine oxidase inhibitors (MAOI)
Moclobemide (MAIO, moclob)
Phenelzine (MAIO, phenel)
Exposure and social skills (EXPO)
Exposure in vivo (EXPO, exposu)
Social skills training (EXPO, social)
Other psychological therapy (OTHR)
Supportive therapy (OTHR, suppor)
Mindfulness (OTHR, mindfu)
Interpersonal psychotherapy (OTHR, interp)
Psychodynamic psychotherapy (PDPT)
Psychodynamic psychotherapy
Group CBT (GCBT)
General, no model specified (GCBT, genera)
Heimberg model (GCBT, Heimbe)
Enhanced CBT (GCBT, enhanc)
Individual CBT (ICBT)
C&W CT shortened sessions (ICBT, short)
Hope, Heimberg & Turk model (ICBT, Hope)
General, no model specified (ICBT, genera)
Clark & Wells Cognitive therapy (ICBT, C&W)
Combined (COMB)
CBT group + Fluoxetine (GCBT & SSRI)
Psychodynamic + Clonazepam (PDPT & BZD)
Paroxetine + Clonazepam (SSRI & BZD)
CBT group + Moclobemide (GCBT & MAOI)
CBT group Heimberg + Phenelzine (GCBT & MAOI)

APPENDIX 12: NETWORK OF SPECIFIC TREATMENTS, RECOVERY

Caption: Network diagram representing direct comparisons of individual interventions restricted to studies reporting recovery, which are arranged by class (as above). The width of lines represents the number of trials making each direct comparison.

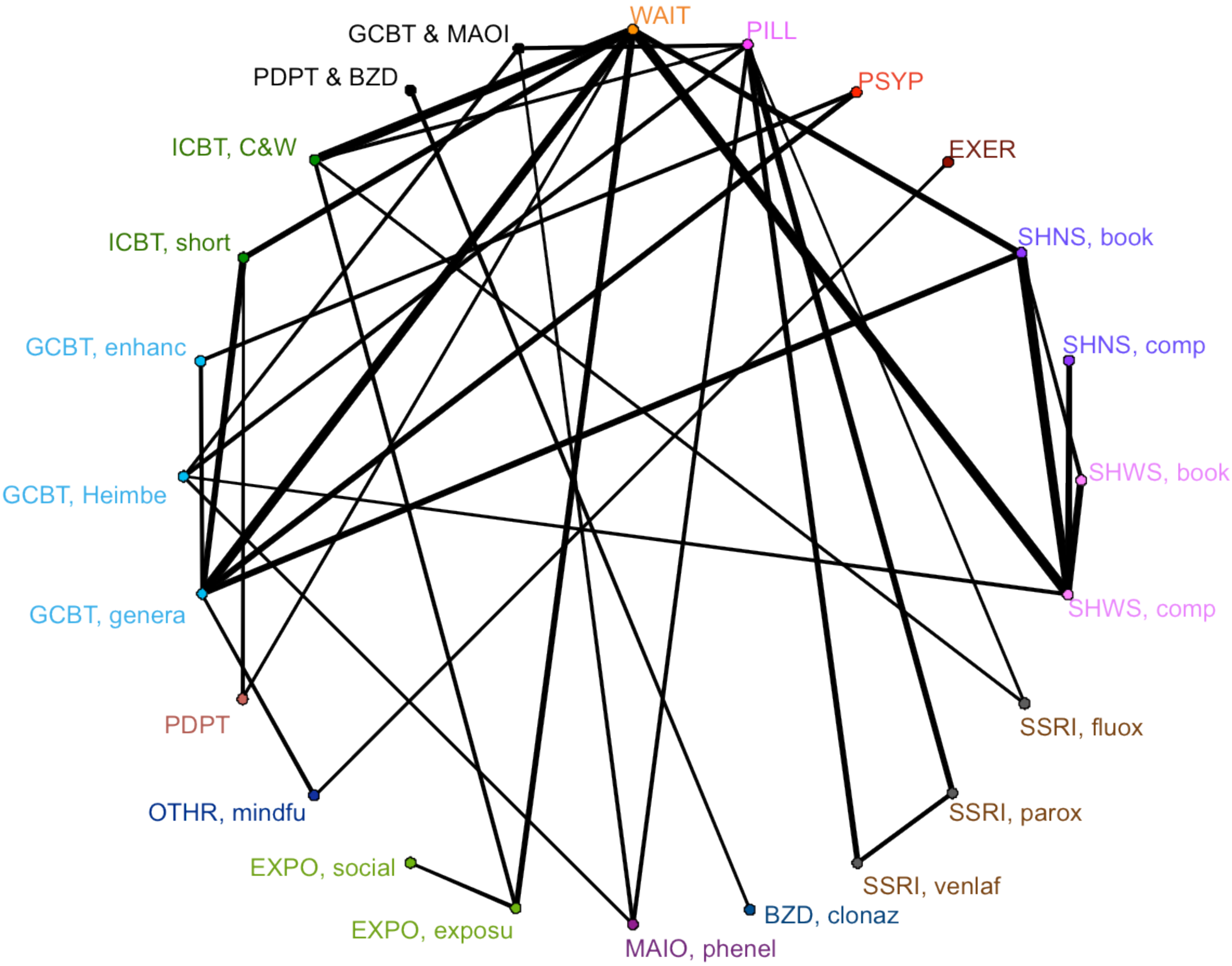

References

1. Brown TA, Di Nardo P, Barlow DH. Anxiety disorders interview schedule for DSM-IV: client interview schedule. Oxford: Oxford University Press, 1994.
2. Davidson JR, Miner CM, De Veaugh-Geiss J, Tupler LA, Colket JT, Potts NL. The Brief Social Phobia Scale: a psychometric evaluation. *Psychol Med* 1997; **27:** 161–66.
3. Davidson JR, Potts NL, Richichi EA, et al. The Brief Social Phobia Scale. *J Clin Psychiatry* 1991; **52** (suppl)**:** 48–51.
4. Guy W. The Clinical Global Impression Scale. ECDEU assessment manual for psychopharmacology-revised. Rockville: US Department of Health, Education and Welfare, ADAMHA, MIMH Psychopharmacology Research Branch, 1976: 218–22.
5. Watson D, Friend R. Measurement of social-evaluative anxiety. *J Consult Clin Psychol* 1969; **33:** 448–57.
6. Marks IM, Mathews AM. Brief standard self-rating for phobic patients. *Behav Res Ther* 1979; **17:** 263–67.
7. Heimberg RG, Horner KJ, Juster HR, et al. Psychometric properties of the Liebowitz Social Anxiety Scale. Psychol Med 1999; 29: 199–212.
8. Fresco DM, Coles ME, Heimberg RG, et al. The Liebowitz Social Anxiety Scale: a comparison of the psychometric properties of self-report and clinician-administered formats. *Psychol Med* 2001; **31:** 1025–35.
9. Baker SL, Heinrichs N, Kim HJ, Hofmann SG. The Liebowitz social anxiety scale as a self-report instrument: a preliminary psychometric analysis. *Behav Res Ther* 2002; **40:** 701–15.
10. Mattick RP, Clarke JC. Development and validation of measures of social phobia scrutiny fear and social interaction anxiety. *Behav Res Ther* 1998; **36:** 455–70.
11. Beidel DC, Turner SM, Stanley MA, Dancu CV. The Social Phobia and Anxiety Inventory: concurrent and external validity. *Behav Ther* 1989; **20:** 417–27.
12. Chinn S. A simple method for converting an odds ratio to effect size for use in meta-analysis. *Stat Med* 2000; **19:** 3127–31.

13. Alden L. Short-term structured treatment for avoidant personality disorder. *J Consult Clin Psychol* 1989; **57:** 756–64.

14. Allsopp LF. Clomipramine and diazepam in the treatment of agoraphobia and social phobia in general practice. *Curr Med Res Opin* 1984; **9:** 64–70.

15. Amir N, Beard C, Taylor CT, et al. Attention training in individuals with generalized social phobia: a randomized controlled trial. *J Consult Clin Psychol* 2009; **77:** 961–73.

16. Amir N, Taylor C. Interpretation training in individuals with generalized social anxiety disorder: a randomized controlled trial. *J Consult Clin Psychol* 2012; **80:** 497–511.

17. Atmaca M, Kuloglu M, Tezcan E, Unal A. Efficacy of citalopram and moclobemide in patients with social phobia: some preliminary findings. *Hum Psychopharmacol* 2002; **17:** 401–05.

18. Barnett SD, Kramer ML, Casat CD, Connor KM, Davidson JR. Efficacy of olanzapine in social anxiety disorder: a pilot study. *J Psychopharmacol* 2002; **16:** 365–68.

19. Beard C, Weisberg RB, Amir N. Combined cognitive bias modification treatment for social anxiety disorder: a pilot trial. *Depress Anxiety* 2011; **28:** 981–88.

20. Boettcher J, Berger T, Renneberg B. Internet-based attention training for social anxiety: a randomized controlled trial. *Cognit Ther Res* 2012; **36:** 522–36.

21. Botella C GM, Garcia PA, Guillen V, Baños RM, Quero S, Alcañiz M. An internet- based self-help treatment for fear of public speaking: a controlled trial. *Cyberpsychol Behav Soc Netw*. 2010; **13:** 407–21.

22. Butler G, Cullington A, Munby M, Amies P, Gelder M. Exposure and anxiety management in the treatment of social phobia. *J Consult Clin Psychol* 1984; **52:** 642–50.

23. Carlbring P, Apelstrand M, Sehlin H, et al. Internet-delivered attention bias modification training in individuals with social anxiety disorder—a double blind randomized controlled trial. *BMC Psychiatry* 2012; **12:** 66.

24. Carlbring P. Treatment of Social Phobia Over the Internet (SOFIE-8) [NCT01312571]. ClinicalTrialsgov: National Institutes of Health; ongoing.

25. Cassin SE, Rector NA. Mindfulness and the attenuation of post-event processing in social phobia: an experimental investigation. *Cogn Behav Ther* 2011; **40:** 267–78.

26. Clark DB, Agras WS. The assessment and treatment of performance anxiety in musicians. *Am J Psychiatry* 1991; **148:** 598–605.

27. Clark DM. Doubling the clinical benefit of each psychotherapy session: a randomised controlled trial of self-study assisted cognitive therapy for social anxiety disorder. [ISRCTN95458747: Internet-based cognitive therapy for social anxiety disorder]. ClinicalTrialsgov: National Institutes of Health; ongoing.

28. Craske MG, Stein MB, Sullivan G, et al. Disorder-specific impact of coordinated anxiety learning and management treatment for anxiety disorders in primary care. *Arch Gen Psychiatry* 2011; **68:** 378–88.

29. Davidson JR, Mangano R. Venlafaxine XR in social anxiety disorder. XIIthWorld Congress of Psychiatry; Yokohama, Japan; Aug 24–29, 2002.

30. D’El Rey GJF, Lacava JPL, Cejkinski A, Mello SL. Cognitive-behavioral group treatment in social phobia: 12-week outcome. *Revista de Psiquiatria Clínica* 2008; **35:** 79–83 (in Portugese).

31. de Oliveira IR, Powell VB, Wenzel A, et al. Efficacy of the trial-based thought record, a new cognitive therapy strategy designed to change core beliefs, in social phobia. *J Clin Pharm Ther* 2012; **37:** 328–34.

32. Donahue CBK. Effect of quetiapine *vs*. placebo on response to two virtual public speaking exposures in individuals with social phobia. *J Anxiety Disord* 2009; **23:** 362–68.

33. Dogaheh ER, Mohammadkhani P, Dolatshahi B. Comparison of group and individual cognitive–behavioral therapy in reducing fear of negative evaluation. *Psychol Rep* 2011; **108:** 955–62.

34. Fahlen T, Nilsson HL, Borg K, Humble M, Pauli U. Social phobia: the clinical efficacy and tolerability of the monoamine oxidase—A and serotonin uptake inhibitor brofaromine. A double-blind placebo-controlled study. *Acta Psychiatr Scand* 1995; **92:** 351–58.

35. Furmark T, Appel L, Michelgard A, et al. Cerebral blood flow changes after treatment of social phobia with the neurokinin-1 antagonist GR205171, citalopram, or placebo. *Biol Psychiatry* 2005; **58:** 132–42.

36. Emmelkamp PM, Mersch PP, Vissia E, van der Helm M. Social phobia: a comparative evaluation of cognitive and behavioral interventions. *Behav Res Ther* 1985; **23:** 365–69.

37. Hayes BB. Comparing the effectiveness of cognitive–behavioral group therapy with and without motivational interviewing at reducing the social anxiety, alcohol consumption, and negative consequences of socially anxious college students. Milwaukee: The University of Wiconsin-Milwaukee, 2006.

38. Heeren A, Reese HE, McNally RJ, Philippot P. Attention training toward and away from threat in social phobia: effects on subjective, behavioral, and physiological measures of anxiety. *Behav Res Ther* 2012; **50:** 30–39.

39. Heideman P. Combining cognitive behavioral therapy with an alcohol intervention to reduce alcohol problems among socially anxious college students. Milwaukee: The University of Wiconsin-Milwaukee, 2008.

40. Hofmann SG. Cognitive mediation of treatment change in social phobia. *J Consult Clin Psychol* 2004; **72:** 393–99.

41. Johnston L, Titov N, Andrews G, Spence J, Dear BF. A RCT of a transdiagnostic internet-delivered treatment for three anxiety disorders: examination of support roles and disorder-specific outcomes. *PloS One* 2011; **6:** e28079.

42. Katzelnick DJ, Kobak KA, Greist JH, Jefferson JW, Mantle JM, Serlin RC. Sertraline for social phobia: a double-blind, placebo-controlled crossover study. *Am J Psychiatry* 1995; **152:** 1368–71.

43. Kobak KA, Taylor LV, Warner G, Futterer R. St. John's wort versus placebo in social phobia: results from a placebo-controlled pilot study. *J Clin Psychopharmacol* 2005; **25:** 51–58.

44. Krylov VI. Pharmacological treatment of social phobia: a controlled study with alprozolam and buspirone: conference abstract. 9th European College of Neuropsychopharmacology Congress; Amsterdam, Netherlands; Sept 21–15, 1996.

45. Lee JY, Choy CH. The effects of the cognitive–behavioral and exposure therapy for social phobia. *Korean Journal of Counseling & Psychotherapy* 1997; **9:** 35–56.

46. Lott M, Greist JH, Jefferson JW, et al. Brofaromine for social phobia: a multicenter, placebo-controlled, double-blind study. *J Clin Psychopharmacol* 1997; **17:** 255–60.

47. Marks IM, Kenwright M, McDonough M, Whittaker M, Mataix-Cols D. Saving clinicians' time by delegating routine aspects of therapy to a computer: a randomized controlled trial in phobia/panic disorder. *Psychol Med* 2004; **34:** 9–17.

48. Mersch PP. The treatment of social phobia: the differential effectiveness of exposure in vivo and an integration of exposure in vivo, rational emotive therapy and social skills training. *Behav Res Ther* 1995; **33:** 259–69.

49. Mortberg E, Karlsson A, Fyring C, Sundin O. Intensive cognitive–behavioral group treatment (CBGT) of social phobia: a randomized controlled study. *J Anxiety Disord* 2006; **20:** 646–60.

50. Muehlbacher M, Nickel MK, Nickel C, et al. Mirtazapine treatment of social phobia in women: a randomized, double-blind, placebo-controlled study. *J Clin Psychopharmacol* 2005; **25:** 580–83.

51. Mulkens S, Bögels SM, de Jong PJ, Louwers J. Fear of blushing: effects of task concentration training versus exposure in vivo on fear and physiology. *J Anxiety Disord* 2001; **15:** 413–32.

52. Neubauer K, Auer Mv, Murray E, Petermann F, Helbig-Lang S, Gerlach AL. Internet-delivered attention modification training as a treatment for social phobia: a randomized controlled trial. *Behav Res Ther* 2013; **51:** 87–97.

53. Norton PJ. A randomized clinical trial of transdiagnostic cognitve–behavioral treatments for anxiety disorder by comparison to relaxation training. *Behav Ther* 2012; **43:** 506–17.

54. Norwegian University of Science and Technology. RCT of cognitive therapy, paroxetine, combined CT and paroxetine and placebo. ClinicalTrialsgov: National Institutes of Health; 2011. P. NCT00184106.

55. Noyes R Jr, Moroz G, Davidson JR, et al. Moclobemide in social phobia: a controlled dose-response trial. *J Clin Psychopharmacol* 1997; **17:** 247–54.

56. Ontiveros Sánchez de la Barquera JA. Estudio controlado doble-ciego con clonazepam y placebo en pacientes con trastorno de ansiedad social. *Salud Mental* 2008; **31:** 299–306.

57. Peng C, Yan L, Ma X, Wu W. Effect of group therapy on social anxiety of college students. *Chinese Mental Health J* 2003; **17:** 229–34.

58. Pollack MH, Stein MB, Ameringen MV. Improving treatment outcomes in pharmacotherapy of generalized social anxiety disorder. ClinicalTrialsgov: National Institutes of Health; 2013. P. NCT00282828.

59. Randall CL, Johnson MR, Thevos AK, et al. Paroxetine for social anxiety and alcohol use in dual-diagnosed patients. *Depress Anxiety* 2001; **14:** 555–62.

60. Ravindran LN, Kim DS, Letamendi AM, Stein MB. A randomized controlled trial of atomoxetine in generalized social anxiety disorder. *J Clin Psychopharmacol* 2009; **29:** 561–64.

61. Schmidt NBR. Attention training for generalized social anxiety disorder. *J Abnorm Psychol* 2009; **118:** 5–14.

62. Schneider AJ, Mataix-Cols D, Marks IM, et al. Internet-guided self-help with or without exposure therapy for phobic and panic disorders. *Psychother Psychosom* 2005; **74:** 154–64.

63. Shaw P. A comparison of three behaviour therapies in the treatment of social phobia. *Br J Psychiatry* 1979; **134:** 620–23.

64. Solyom CS. Phenelzine and exposure in the treatment of phobias. *Biol Psychiatry*1981; **16:** 239–47.

65. Taylor S, Woody S, Koch WJ, McLean P, Paterson RJ, Anderson KW. Cognitive restructuring in the treatment of social phobia. Efficacy and mode of action. *Behav Modif* 1997; **21:** 487–511.

66. Tejedor AC, Martinez LdN. Social skills training: a comparative study. *Analisis y Modificacion de Conducta* 1996; **22:** 709–27 (in Spanish).

67. Taylor CT, Alden LE. Safety behaviors and judgmental biases in social anxiety disorder. *Behav Res Ther* 2010; **48:** 226–37.

68. Turner SM, Beidel DC, Jacob RG. Social phobia: a comparison of 37ehaviour therapy and atenolol. *J Consult Clin Psychol* 1994; **62:** 350–58.

69. Tyrer P, Candy J, Kelly D. A study of the clinical effects of phenelzine and placebo in the treatment of phobic anxiety. *Psychopharmacologia* 1973; **32:** 237–54.

70. Vaishnavi S, Alamy S, Zhang W, Connor KM, Davidson JR. Quetiapine as monotherapy for social anxiety disorder: a placebo-controlled study. *Prog Neuropsychopharmacol Biol Psychiatry* 2007; **31:** 1464–69.

71. Van Ameringen M, Mancini C, Oakman J, et al. Nefazodone in the treatment of generalized social phobia: a randomized, placebo-controlled trial. *J Clin Psychiatry* 2007; **68:** 288–95.

72. Ameringen MV. Sertraline in generalized social phobia with co-occurring anxiety and mood disorders [NCT00182533]. ClinicalTrialsgov: National Institutes of Health; ongoing.

73. van Vliet IM, den Boer JA, Westenberg HG. Psychopharmacological treatment of social phobia: clinical and biochemical effects of brofaromine, a selective MAO-A inhibitor. *Eur Neuropsychopharmacol* 1992; **2:** 21–29.

74. van Vliet IM, den Boer JA, Westenberg HG, Pian KL. Clinical effects of buspirone in social phobia: a double-blind placebo-controlled study. *J Clin Psychiatry* 1997; **58:** 164–68.

75. Versiani MN. Double-blind placebo controlled trial with bromazepam in social phobia. *J Bras Psiquiatr* 1997; **46:** 167–71.

76. Yang K, Yang M, Liu H. Comparative study of the effects of accompanied systemic desensitization in the treatment of social phobia. *Chinese Mental Health J* 1999; **13:** 238–39 (in Chinese).

77. Yoshinaga N, Niitsu T, Hanaoka H, et al. Strategy for treating selective serotonin reuptake inhibitor-resistant social anxiety disorder in the clinical setting: a randomised controlled trial protocol of cognitive behavioural therapy in combination with conventional treatment. *BMJ Open* 2013; **3:** e002242.

78. Zhang Y, Yang D, Xiao Z, et al. The role of Taoist cognitive psychotherapy in the treatment of anxiety disorder. *Chinese Mental Health J* 2000; **14:** 62–63 (in Chinese).

79. Zhang G, Wei HY, Zeng ZX, et al. A double-blinded study of paroxetine and alprazolam in the treatment of social phobia. *Shandong Archives of Psychiatry* 2003; **16:** 144–46.

80. Abramowitz JS, Moore E. Self-help cognitive-behavioral therapy with minimal therapist contact for social phobia: a controlled trial. *J Behav Ther Exp Psychiatry* 2009; **40:** 98–105.

81. Alden LE, Taylor CT. Relational treatment strategies increase social approach behaviors in patients with generalized social anxiety disorder. *J Anxiety Disord* 2011; **25:** 309–18.

82. Allgulander C. Efficacy of paroxetine in social phobia—a single-center double-blind study of 96 symptomatic volunteers randomized to treatment with paroxetine 20–50 mg or placebo for 3 months. 11th European College of Neuropsychopharmacology Congress; Paris, France; Oct 31 to Nov 4, 1998.

83. Allgulander C. Paroxetine in social anxiety disorder: a randomized placebo-controlled study. *Acta Psychiatr Scand* 1999; **100:** 193–98.

84. Allgulander C, Nilsson B. A prospective study of 86 new patients with social anxiety disorder. *Acta Psychiatr Scand* 2001; **103:** 447–52.

85. Allgulander C, Mangano R, Zhang J, et al. Efficacy of venlafaxine ER in patients with social anxiety disorder: a double-blind, placebo-controlled, parallel-group comparison with paroxetine. *Hum Psychopharmacol* 2004; **19:** 387–96.

86. Andersson G, Carlbring P, Holmström A, et al. Internet-based self-help with therapist feedback and in vivo group exposure for social phobia: a randomized controlled trial. *J Consult Clin Psychol* 2006; **74:** 677–86.

87. Andersson G, Carlbring P, Furmark T. Therapist experience and knowledge acquisition in internet-delivered CBT for social anxiety disorder: a randomized controlled trial. *PloS One* 2012; **7:** e37411.

88. Andrews G, Davies M, Titov N. Effectiveness randomized controlled trial of face to face versus Internet cognitive behaviour therapy for social phobia. *Aust NZ J Psychiatry* 2011; **45:** 337–40.

89. Asakura S, Tajima O, Koyama T. Fluvoxamine treatment of generalized social anxiety disorder in Japan: a randomized double-blind, placebo-controlled study. *Int J Neuropsychopharmacol* 2007; **10:** 263–74.

90. Baldwin DS, Bobes J, Stein DJ, Scharwächter I, Faure M. Paroxetine in social phobia/social anxiety disorder. Randomised, double-blind, placebo-controlled study. Paroxetine Study Group. *Br J Psychiatry* 1999; **175:** 120–26.

91. Stein DJB. A double-blind placebo-controlled trial of paroxetine in the management of social phobia (social anxiety disorder) in South Africa. *S Afr Med J* 1999; **89:** 402–06.

92. Stein DJS. The selective serotonin reuptake inhibitor paroxetine is effective in more generalized and in less generalized social anxiety disorder. *Psychopharmacology (Berl)* 2001; **158:** 267–72.

93. Berger T, Hohl E, Caspar F. Internet-based treatment for social phobia: a randomized controlled trial. *J Clin Psychology* 2009; **65:** 1021–35.

94. Berger T, Caspar F, Richardson R, Kneubuhler B, Sutter D, Andersson G. Internet-based treatment of social phobia: a randomized controlled trial comparing unguided with two types of guided self-help. *Behav Res Ther* 2011; **49:** 158–69.

95. Bjornsson AS, Bidwell LC, Brosse AL, et al. Cognitive–behavioral group therapy versus group psychotherapy for social anxiety disorder among college students: a randomized controlled trial. *Depress Anxiety* 2011; **28:** 1034–42.

96. Blanco C, Heimberg RG, Schneier FR, et al. A placebo-controlled trial of phenelzine, cognitive behavioral group therapy, and their combination for social anxiety disorder. *Arch Gen Psychiatry* 2010; **67:** 286–95.

97. Blomhoff S, Haug TT, Hellstrom K, et al. Randomised controlled general practice trial of sertraline, exposure therapy and combined treatment in generalised social phobia. *Br J Psychiatry* 2001; **179:** 23–30.

98. Borgeat F, Stankovic M, Khazaal Y, et al. Does the form or the amount of exposure make a difference in the cognitive–behavioral therapy treatment of social phobia? *J Nerv Ment Dis* 2009; **197:** 507–13.

99. Bowler JO, Mackintosh B, Dunn BD, Mathews A, Dalgleish T, Hoppitt L. A comparison of cognitive bias modification for interpretation and computerized cognitive behavior therapy: effects on anxiety, depression, attentional control, and interpretive bias. *J Consult Clin Psychol* 2012; **80:** 1021–33.

100. Burrows GE. Moclobemide in social phobia. A double-blind, placebo-controlled clinical study. *Eur Arch Psychiatry Clin Neurosci* 1997; **247:** 71–80.

101. Almlov J, Carlbring P, Kallqvist K, Paxling B, Cuijpers P, Andersson G. Therapist effects in guided internet-delivered CBT for anxiety disorders. *Behav Cogn Psychother* 2011; **39:** 311–22.

102. Carlbring P, Gunnarsdóttir M, Hedensjö L, Andersson G, Ekselius L, Furmark T. Treatment of social phobia: randomised trial of internet-delivered cognitive-behavioural therapy with telephone support. *Br J Psychiatry* 2007; **190:** 123–28.

103. Carlbring P, Nordgren LB, Furmark T, Andersson G. Long-term outcome of Internet-delivered cognitive–behavioural therapy for social phobia: a 30-month follow-up. *Behav Res Ther* 2009; **47:** 848–50.

104. Nordgreen T, Havik OE, Ost LG, Furmark T, Carlbring P, Andersson G. Outcome predictors in guided and unguided self-help for social anxiety disorder. *Behav Res Ther* 2012; **50:** 13–21.

105. Sun CY, Hye KJ. The efficacy of bibliotherapy for social phobia. *Brief Treatment and Crisis Intervention*. 2008; **8:** 390–401.

106. Clark DM. A controlled trial of cognitive therapy and pharmacotherapy in the treatment of social phobia. National Research Register, 1998.

107. Clark DM, Ehlers A, McManus F, et al. Cognitive therapy versus fluoxetine in generalized social phobia: a randomized placebo-controlled trial. *J Consult Clin Psychol* 2003; **71:** 1058–67.

108. Clark DM. Cognitive therapy versus exposure and applied relaxation in social phobia: a randomized controlled trial. *J Consult Clin Psychol* 2006; **74:** 568–78.

109. McManus F, Clark DM, Grey N, et al. A demonstration of the efficacy of two of the components of cognitive therapy for social phobia. *J Anxiety Disord* 2009; **23:** 496–503.

110. Clark DM, Wild J, Grey N, et al. Self-study enhances the effects of cognitive therapy for social anxiety disorder: a randomized controlled Trial (ISRCTN11178360). 2012.

111. Cottraux J, Note I, Albuisson E, et al. Cognitive behavior therapy versus supportive therapy in social phobia: a randomized controlled trial. *Psychother Psychosom* 2000; **69:** 137–46.

112. Davidson JR, Potts N, Richichi E, et al. Treatment of social phobia with clonazepam and placebo. *J Clin Psychopharmacol* 1993; **13:** 423–28.

113. Sutherland SM, Tupler LA, Colket JT, Davidson JR. A 2-year follow-up of social phobia. Status after a brief medication trial. *J Nerv Ment Dis* 1996; **184:** 731–38.

114. Davidson J, Yaryura-Tobias J, DuPont R, et al. Fluvoxamine-controlled release formulation for the treatment of generalized social anxiety disorder. *J Clin Psychopharmacol* 2004; **24:** 118–25.

115. Davidson JR, Hemby LW, Barbato L, van-der-Hoop RG. Fluvoxamine controlled release for the treatment of generalized social anxiety disorder. 39th Annual Meeting of the American College of Neuropsychopharmacology; San Juan, Puerto Rico; Dec 10–14. 2000: 161.

116. Owen RT. Controlled-release fluvoxamine in obsessive-compulsive disorder and social phobia. *Drugs Today (Barc)* 2008; **44:** 887–93.

117. Davidson JR, Foa EB, Huppert JD, et al. Fluoxetine, comprehensive cognitive behavioral therapy, and placebo in generalized social phobia. *Arch Gen Psychiatry* 2004; **61:** 1005–13.

118. Ledley DRH. Impact of depressive symptoms on the treatment of generalized social anxiety disorder. *Depress Anxiety* 2005; **22:** 161–67.

119. Emmelkamp PM, Benner A, Kuipers A, Feiertag GA, Koster HC, van Apeldoorn FJ. Comparison of brief dynamic and cognitive–behavioural therapies in avoidant personality disorder. *Br J Psychiatry* 2006; **189:** 60–64.

120. Feltner DE, Liu-Dumaw M, Schweizer E, Bielski R. Efficacy of pregabalin in generalized social anxiety disorder: results of a double-blind, placebo-controlled, fixed-dose study. *Int Clin Psychopharmacol*2011; **26:** 213–20.

121. Furmark TT. Common changes in cerebral blood flow in patients with social phobia treated with citalopram or cognitive–behavioral therapy. *Arch Gen Psychiatry* 2002; **59:** 425–33.

122. Furmark T, Appel L, Michelgård A, et al. Cerebral blood flow changes after treatment of social phobia with the neurokinin-1 antagonist GR205171, citalopram, or placebo. *Biol Psychiatry*2005; **58:** 132–42.

123. Furmark T, Carlbring P, Hedman E, et al. Guided and unguided self-help for social anxiety disorder: randomised controlled trial. *Br J Psychiatry* 2009; **195:** 440–47.

124. Hedman E, Furmark T, Carlbring P, et al. A 5-year follow-up of internet-based cognitive behavior therapy for social anxiety disorder. *J Med Internet Res* 2011; **13:** e39.

125. Gelernter CS. A comparison of cognitive–behavioral and pharmacological treatments for social phobia. Dissertation Abstracts International. 1990; **50**: 3156.

126. Gelernter CS, Uhde TW, Cimbolic P, et al. Cognitive–behavioral and pharmacological treatments of social phobia: a controlled study. *Arch Gen Psychiatry* 1991; **48:** 938–45.

127. Goldin PR, Ziv M, Jazaieri H, et al. Cognitive reappraisal self-efficacy mediates the effects of individual cognitive–behavioral therapy for social anxiety disorder. *J Consult Clin Psychol* 2012; **80:** 1034–40.

128. Gruber K, Moran PJ, Roth WT, Taylor CB. Computer-assisted cognitive behavioral group therapy for social phobia. *Behav Ther* 2001; **32:** 155–65.

129. GlaxoSmithKline. A randomized, double-blind, parallel-group, placebo-controlled, forced-dose titration study evaluating the efficacy and safety of a New Chemical Entity (NCE) and paroxetine in subjects with social anxiety disorder. GSKClinical Study Register [wwwgskclinicalstudyregistercom]. 2006.

130. Hedman E, Andersson G, Ljotsson B, et al. Internet-based cognitive behavior therapy vs. cognitive behavioral group therapy for social anxiety disorder: a randomized controlled non-inferiority trial. *PloS One* 2011; **6:** e18001.

131. Hedman E, Andersson E, Ljotsson B, et al. Clinical and genetic outcome determinants of internet- and group-based cognitive behavior therapy for social anxiety disorder. *Acta Psychiatr Scand* 2012; **126:** 126–36.

132. Heimberg RG, Dodge CS, Hope DA, Kennedy CR, Zollo LJ, Becker RE. Cognitive behavioral group treatment for social phobia: comparison with a credible placebo control. *Cognit Ther Res* 1990; **14:** 1–23.

133. Heimberg RG, Salzman DG, Holt CS, Blendell KA. Cognitive–behavioral group treatment for social phobia: effectiveness at five-year followup. *Cognit Ther Res* 1993; **17:** 325–39.

134. Heimberg RG, Liebowitz MR, Hope DA, et al. Cognitive behavioral group therapy *vs* phenelzine therapy for social phobia 12-week outcome. *Arch Gen Psychiatry* 1998; **55:** 1133–41.

135. Liebowitz MRH. Cognitive–behavioral group therapy versus phenelzine in social phobia: long term outcome. *Depress Anxiety* 1999; **10:** 89–98.

136. Thyer BA. Cognitive behavioral group therapy and phenelzine both effective in social phobia. *West J Med* 1999; **171:** 240.

137. Herbert JD, Rheingold AA, Gaudiano BA, Myers VH. Standard versus extended cognitive behavior therapy for social anxiety disorder: a randomized-controlled trial. *Behav Cogn Psychother* 2004; **32:** 131–47.

138. Herbert JD, Gaudiano BA, Rheingold AA, Myers VH, Dalrymple K, Nolan EM. Social skills training augments the effectiveness of cognitive behavioral group therapy for social anxiety disorder. *Behav Ther* 2005; **36:** 125–38.

139. Hope DA, Heimberg RG, Bruch MA. Dismantling cognitive–behavioral group therapy for social phobia. *Behav Res Ther* 1995; **33:** 637–50.

140. Jazaieri H, Goldin P, Werner K, Ziv M, Gross J. A randomized trial of MBSR versus aerobic exercise for social anxiety disorder. *J Clin Psychol* 2012; **68:** 715–31.

141. Kasper S, Loft H, Smith JR. Escitalopram is efficacious and well tolerated in the treatment of social anxiety disorder. 155th Annual Meeting of the American Psychiatric Association; Philadelphia, PA, USA; May 18–23, 2002: NR491.

142. Kasper S, Stein DJ, Loft H, Nil R. Escitalopram in the treatment of social anxiety disorder: randomised, placebo-controlled, flexible-dosage study. *Br J Psychiatry* 2005; **186:** 222–26.

143. Knijnik DZ, Kapczinski F, Chachamovich E, Margis R, Eizirik CL. Psychodynamic group treatment for generalized social phobia. *Revista Brasileira de Psiquiatria* 2004; **26:** 77–81.

144. Knijnik DZ, Blanco C, Salum GA, et al. A pilot study of clonazepam versus psychodynamic group therapy plus clonazepam in the treatment of generalized social anxiety disorder. *Eur Psychiatry* 2008; **23:** 567–74.

145. Kobak KA, Greist JH, Jefferson JW, Katzelnick DJ. Fluoxetine in social phobia: a double-blind, placebo-controlled pilot study. *J Clin Psychopharmacol* 2002; **22:** 257–62.

146. Koszycki D, Benger M, Shlik J, Bradwejn J. Randomized trial of a meditation-based stress reduction program and cognitive behavior therapy in generalized social anxiety disorder. *Behav Res Ther* 2007; **45:** 2518–26.

147. Lader M, Stender K, Bürger V, Nil R. Efficacy and tolerability of escitalopram in 12- and 24-week treatment of social anxiety disorder: randomised, double-blind, placebo-controlled, fixed-dose study. *Depress Anxiety* 2004; **19:** 241–48.

148. Stein DJ, Andersen EW, Lader M. Escitalopram versus paroxetine for social anxiety disorder: an analysis of efficacy for different symptom dimensions. *Eur Neuropsychopharmacol* 2006; **16:** 33–38.

149. Ledley DR, Heimberg RG, Hope DA, et al. Efficacy of a manualized and workbook-driven individual treatment for social anxiety disorder. *Behav Ther* 2009; **40:** 414–24.

150. Leichsenring F, Salzer S, Beutel ME, et al. Psychodynamic therapy and cognitive-behavioral therapy in social anxiety disorder: a multicenter randomized controlled trial. *Am J Psychiatry* 2013; **170:** 759–67.

151. Lepola U, Bergtholdt B, St Lambert J, Davy KL, Ruggiero L. Controlled-release paroxetine in the treatment of patients with social anxiety disorder. *J Clin Psychiatry* 2004; **65:** 222–29.

152. Liebowitz MR, Schneier F, Campeas R, et al. Phenelzine and atenolol in social phobia. *Psychopharmacol Bull* 1990; **26:** 123–25.

153. Liebowitz MRG. Pharmacotherapy of social phobia: an interim report of a placebo-controlled comparison of phenelzine and atenolol. *J Clin Psychiatry* 1988; **49:** 252–57.

154. Liebowitz MR, Schneier F, Campeas R, et al. Phenelzine *vs* atenolol in social phobia. A placebo-controlled comparison. *Arch Gen Psychiatry* 1992; **49:** 290–300.

155. GlaxoSmithKline. A randomized, double-blind, fixed dose comparison of 20, 40, and 60mg daily of paroxetine and placebo in the treatment of generalized social phobia. GSKClinical Study Register [wwwgskclinicalstudyregistercom]. 1997.

156. Liebowitz MR, Stein MB, Tancer M, Carpenter D, Oakes R, Pitts CD. A randomized, double-blind, fixed-dose comparison of paroxetine and placebo in the treatment of generalized social anxiety disorder. *J Clin Psychiatry* 2002; **63:** 66–74.

157. Liebowitz MR, DeMartinis NA, Weihs K, et al. Efficacy of sertraline in severe generalized social anxiety disorder: results of a double-blind, placebo-controlled study. *J Clin Psychiatry* 2003; **64:** 785–92.

158. Connor KM, Davidson JR, Chung H, Yang R, Clary CM. Multidimensional effects of sertraline in social anxiety disorder. *Depress Anxiety* 2006; **23:** 6–10.

159. Liebowitz M, DeMartinis N, Weihs K, Chung H, Clary C. Results from a randomized, double-blind, multicenter trial of sertaline in the treatment of moderate-to-severe social phobia (social anxiety disorder). *Eur Neuropsychopharmacol* 2002; **12:** S352.

160. Liebowitz M, Mangano RM. Efficacy of venlafaxine xr in generalized social anxiety disorder. *Int J Neuropsychopharmacol* 2002; **5:** 211.

161. Liebowitz MR, Mangano RM, Bradwejn J, Asnis G. A randomized controlled trial of venlafaxine extended release in generalized social anxiety disorder. *J Clin Psychiatry* 2005; **66:** 238–47.

162. Liebowitz MR, Gelenberg AJ, Munjack D. Venlafaxine extended release vs placebo and paroxetine in social anxiety disorder. *Arch Gen Psychiatry* 2005; **62:** 190–98.

163. Lipsitz JD, Gur M, Vermes D, et al. A randomized trial of interpersonal therapy versus supportive therapy for social anxiety disorder. *Depress Anxiety* 2008; **25:** 542–53.

164. Mattick RP, Peters L. Treatment of severe social phobia: effects of guided exposure with and without cognitive restructuring. *J Consult Clin Psychol* 1988; **56:** 251–60.

165. Mattick RP, Peters L, Clarke JC. Exposure and cognitive restructuring for social phobia: a controlled study. *Behav Ther* 1989; **20:** 3–23.

166. McEvoy PM, Perini SJ, McEvoy PM, Perini SJ. Cognitive behavioral group therapy for social phobia with or without attention training: a controlled trial. *J Anxiety Disord* 2009; **23:** 519–28.

167. Morgan H, Raffle C. Does reducing safety behaviours improve treatment response in patients with social phobia? *Aust NZ J Psychiatry* 1999; **33:** 503–10.

168. Mortberg E, Clark DM, Bejerot S. Intensive group cognitive therapy and individual cognitive therapy for social phobia: sustained improvement at 5-year follow-up. *J Anxiety Disord* 2011; **25:** 994–1000.

169. Mortberg E, Clark DM, Sundin O, Wistedt AA. Intensive group cognitive treatment and individual cognitive therapy *vs*. treatment as usual in social phobia: a randomized controlled trial. *Acta Psychiatr Scand* 2007; **115:** 142–54.

170. Mortberg EB. Temperament and character dimensions in patients with social phobia: Patterns of change following treatments? *Psychiatry Res* 2007; **152:** 81–90.

171. Munjack DJ, Baltazar PL, Bohn PB, Cabe DD, Appleton AA. Clonazepam in the treatment of social phobia: a pilot study. *J Clin Psychiatry* 1990; **51**(5 suppl)**:** 35–40.

172. Oosterbaan DB, van Balkom AJLM, Spinhoven P, van Oppen P, van Dyck R. Cognitive therapy versus moclobemide in social phobia: a controlled study. *Clin Psychol Psychother* 2001; **35:** 889–900.

173. Otto MW, Pollack MH, Gould RA, Worthington JJ, McArdle ET, Rosenbaum JF. A comparison of the efficacy of clonazepam and cognitive–behavioral group therapy for the treatment of social phobia. *J Anxiety Disord* 2000; **14:** 345–58.

174. Pande AC, Davidson JR, Jefferson JW, et al. Treatment of social phobia with gabapentin: a placebo-controlled study. *J Clin Psychopharmacol* 1999; **19:** 341–48.

175. Feltner DE, Pollack MH, Davidson JR, et al. A placebo-controlled study of pregabalin in social phobia (social anxiety disorder). *Int J Neuropsychopharmacol* 2000; **3:** 284.

176. Pande AC, Feltner DE, Jefferson JW, et al. Efficacy of the novel anxiolytic pregabalin in social anxiety disorder: a placebo-controlled, multicenter study. *J Clin Psychopharmacol* 2004; **24:** 141–49.

177. Pfizer. A 10-week, randomized, double-blind, placebo-controlled study of paroxetine and pregabalin in patients with social phobia (1008-081 and 1008-153). Web Synopsis Protocol 1008-081/153 – 21 June 2007 – Final; 2007.

178. Piet J, Hougaard E, Hecksher MS, Rosenberg NK. A randomized pilot study of mindfulness-based cognitive therapy and group cognitive–behavioral therapy for young adults with social phobia. *Scand J Psychol* 2010; **51:** 403–10.

179. Prasetyo J, Horacek J, Paskova B, Praskova H. Moclobemide and/or CBT in the treatment of social phobia: 6-months study. XII World Congress of Psychiatry; Yokohama, Japan; Aug 24–9, 2002.

180. Prasko J, Horacek J, Paskova B. Moclobemide and/or CBT in the treatment of social phobia. 32nd Congress of the British Association for Behavioural and Cognitive Psychotherapies; Manchester, UK; Sept 7–11, 2004: 78.

181. Prasko J, Kosova J, Klaschka J, Seifertova D, Paskova H. Pharmacotherapy and psychotherapy in the treatment of social phobia – Controlled study. XI World Congress of Psychiatry; Hamburg, Germany; Aug 6–11, 1999: abstracts volume II: 94.

182. Prasko JD. Moclobemide and cognitive behavioral therapy in the treatment of social phobia. A six-month controlled study and 24 months follow up. *Neuro Endocrinol Lett* 2006; **27:** 473–81.

183. Prasko JK. Pharmacotherapy and/or cognitive–behavioral therapy in the treatment of social phobia—control study with two year follow up. *Ceska a Slovenska Psychiatrie* 2003; **99**(2 suppl)**:** 106–08.

184. Rapee RM, Abbott MJ, Baillie AJ, Gaston JE. Treatment of social phobia through pure self-help and therapist-augmented self-help. *Br J Psychiatry* 2007; **191:** 246–52.

185. Rapee RM, Gaston JE, Abbott MJ. Testing the efficacy of theoretically derived improvements in the treatment of social phobia. *J Consult Clin Psychol* 2009; **77:** 317–27.

186. Rickels K, Mangano R, Khan A. A double-blind, placebo-controlled study of a flexible dose of venlafaxine ER in adult outpatients with generalized social anxiety disorder. *J Clin Psychopharmacol* 2004; **24:** 488–96.

187. Robillard G, Bouchard S, Dumoulin S, Guitard T, Klinger E. Using virtual humans to alleviate social anxiety: preliminary report from a comparative outcome study. *Stud Health Technol Inform* 2010; **154:** 57–60.

188. Salaberria K, Echeburua E. Long-term outcome of cognitive therapy's contribution to self-exposure in vivo to the treatment of generalized social phobia. *Behav Modif* 1998; **22:** 262–84.

189. Schneier FR, Goetz D, Campeas R, Fallon B, Marshall R, Liebowitz MR. Placebo-controlled trial of moclobemide in social phobia. *Br J Psychiatry* 1998; **172:** 70–77.

190. Duffett R. Moclobemide in social phobia. *Br J Psychiatry* 1998; **172:** 451–52.

191. Schutters SI, Van Megen HJ, Van Veen JF, Denys DA, Westenberg HG. Mirtazapine in generalized social anxiety disorder: a randomized, double-blind, placebo-controlled study. *Int Clin Psychopharmacol*2010; **25:** 302–04.

192. Seedat S, Stein MB. Double-blind, placebo-controlled assessment of combined clonazepam with paroxetine compared with paroxetine monotherapy for generalized social anxiety disorder. *J Clin Psychiatry* 2004; **65:** 244–48.

193. Smits JA, Powers MB, Buxkamper R, Telch MJ. The efficacy of videotape feedback for enhancing the effects of exposure-based treatment for social anxiety disorder: a controlled investigation. *Behav Res Ther* 2006; **44:** 1773–85.

194. Stangier U, Heidenreich T, Peitz M, Lauterbach W, Clark DM. Cognitive therapy for social phobia: individual versus group treatment. *Behav Res Ther* 2003; **41:** 991–1007.

195. Stangier U, Schramm E, Heidenreich T, Berger M, Clark DM. Cognitive therapy *vs* interpersonal psychotherapy in social anxiety disorder: a randomized controlled trial. *Arch Gen Psychiatry* 2011; **68:** 692–700.

196. Stein MB, Liebowitz MR, Lydiard RB, Pitts CD, Bushnell W, Gergel I. Paroxetine treatment of generalized social phobia (social anxiety disorder): a randomized controlled trial. *JAMA* 1998; **280:** 708–13.

197. Stein MB, Fyer AJ, Davidson JR, Pollack MH, Wiita B. Fluvoxamine treatment of social phobia (social anxiety disorder): a double-blind, placebo-controlled study. *Am J Psychiatry* 1999; **156:** 756–60.

198. Stein DJ, Cameron A, Amrein R, Montgomery SA. Moclobemide is effective and well tolerated in the long-term pharmacotherapy of social anxiety disorder with or without comorbid anxiety disorder. *Int Clin Psychopharmacol* 2002; **17:** 161–70.

199. Stein MB, Pollack MH, Bystritsky A, Kelsey JE, Mangano RM. Efficacy of low and higher dose extended-release venlafaxine in generalized social anxiety disorder: a 6-month randomized controlled trial. *Psychopharmacology (Berl)* 2005; **177:** 280–88.

200. Stravynski A, Arbel N, Bounader J, et al. Social phobia treated as a problem in social functioning: a controlled comparison of two behavioural group approaches. *Acta Psychiatr Scand* 2000; **102:** 188–98.

201. Tauscher J, Kielbasa W, Iyengar S, et al. Development of the 2nd generation neurokinin-1 receptor antagonist LY686017 for social anxiety disorder. *Eur Neuropsychopharmacol* 2010; **20:** 80–87.

202. Titov N, Andrews G, Johnston L, Schwencke G, Choi I. Shyness programme: longer term benefits, cost-effectiveness, and acceptability. *Aust NZ J Psychiatry* 2009; **43:** 36–44.

203. Titov N, Andrews G, Schwencke G, Drobny J, Einstein D. Shyness 1: distance treatment of social phobia over the Internet. *Aust NZ J Psychiatry* 2008; **42:** 585–94.

204. Titov N, Gibson M, Andrews G, McEvoy P. Internet treatment for social phobia reduces comorbidity. *Aust NZ J Psychiatry* 2009; **43:** 754–59.

205. Titov N, Andrews G, Schwencke G. Shyness 2: treating social phobia online: replication and extension. *Aust NZ J Psychiatry* 2008; **42:** 595–605.

206. Titov N, Andrews G, Choi I, Schwencke G, Mahoney A. Shyness 3: randomized controlled trial of guided versus unguided Internet-based CBT for social phobia. *Aust NZ J Psychiatry* 2008; **42:** 1030–40.

207. Titov N, Andrews G, Schwencke G, Solley K, Johnston L, Robinson E. An RCT comparing effect of two types of support on severity of symptoms for people completing Internet-based cognitive behaviour therapy for social phobia. *Aust NZ J Psychiatry* 2009; **43:** 920–26.

208. Titov N, Andrews G, Choi I, Schwencke G, Johnston L. Randomized controlled trial of web-based treatment of social phobia without clinician guidance. *Aust NZ J Psychiatry* 2009; **43:** 913–19.

209. Titov N, Andrews G, Schwencke G, Robinson E, Peters L, Spence J. Randomized controlled trial of Internet cognitive behavioural treatment for social phobia with and without motivational enhancement strategies. *Aust NZ J Psychiatry* 2010; **44:** 938–45.

210. Van Ameringen M, Oakman J, Mancini C, Pipe B, Chung H. Predictors of response in generalized social phobia: effect of age of onset. *J Clin Psychopharmacol* 2004; **24:** 42–48.

211. Van Ameringen MA, Lane RM, Walker JR, et al. Sertraline treatment of generalized social phobia: a 20-week, double-blind, placebo-controlled study. *Am J Psychiatry* 2001; **158:** 275–81.

212. Ameringen MV, Mancini C, Chung H, Batzar E, Yang R. Predictors of high endpoint response in patients treated with sertraline for moderate to severe generalized social phobia. *Int J Neuropsychopharmacol* 2002; **5:** 213.

213. Walker JR, Van Ameringen MA, Swinson R, et al. Prevention of relapse in generalized social phobia: results of a 24-week study in responders to 20 weeks of sertraline treatment. *J Clin Psychopharmacol* 2000; **20:** 636–44.

214. Van-Ameringen MA, Swinson R, Walker JR, Lane RM. A placebo-controlled study of sertraline in generalized social phobia. *Eur Neuropsychopharmacol* 2000; **10:** S335.

215. den Boer JA, van Vliet I, Westenberg HG. A double-blind placebo controlled study of fluvoxamine in social phobia. *Clin Neuropharmacol* 1992; **15:** 91B.

216. van Vliet IM, den Boer JA, Westenberg HG. Psychopharmacological treatment of social phobia; a double blind placebo controlled study with fluvoxamine. *Psychopharmacology (Berl)* 1994; **115:** 128–34.

217. Versiani M, Nardi AE, Mundim FD, Alves AB, Liebowitz MR, Amrein R. Pharmacotherapy of social phobia. A controlled study with moclobemide and phenelzine. *Br J Psychiatry* 1992; **161:** 353–60.

218. Stein DJW. Fluvoxamine CR in the long-term treatment of social anxiety disorder: The 12- to 24-week extension phase of a multicentre, randomized, placebo-controlled trial. *Int J Neuropsychopharmacol* 2003; **6:** 317–23.

219. Westenberg HG, Stein DJ, Yang H, Li D, Barbato LM. A double-blind placebo-controlled study of controlled release fluvoxamine for the treatment of generalized social anxiety disorder. *J Clin Psychopharmacol* 2004; **24:** 49–55.

220. Willutzki U, Neumann B, Haas H, Koban C, Schulte D. Zur Psychotherapie sozialer Angste: Kognitive Verhaltenstherapie im Vergleich zu einem kombiniert ressourcenorientierten Vorgehen. *Zeitschrift fur Klinische Psychologie und Psychotherapie* 2004; **33:** 42–50 (in German).

221. Willutzki U, Teismann T, Schulte D. Zur Psychotherapie sozialer Angste: Veranderungen des Wohlbefindens bei kognitiver Verhaltenstherapie im Vergleich zu einem kombiniert-ressourcenorientierten Vorgehen. *Zeitschrift fur Klinische Psychologie und Psychotherapie* 2011; **40:** 53–58 (in German).

222. Willutzki U, Teismann T, Schulte D. Psychotherapy for social anxiety disorder: long-term effectiveness of resource-oriented cognitive–behavioral therapy and cognitive therapy in social anxiety disorder. *J Clin Psychology* 2012; **68:** 581–91.

223. Wong DFK, Sun SYK. A preliminary study of the efficacy of group cognitive–behavioural therapy for people with social anxiety in Hong Kong. *Hong Kong J Psychiatry* 2007; **16:** 50–56.

224. Zhang W, Connor KM, Davidson JR. Levetiracetam in social phobia: a placebo controlled pilot study. *J Psychopharmacol* 2005; **19:** 551–53.
